# Integrin signaling in pluripotent cells acts as a gatekeeper of mouse germline entry

**DOI:** 10.1126/sciadv.adk2252

**Published:** 2024-09-04

**Authors:** Aly Makhlouf, Anfu Wang, Nanami Sato, Viviane S. Rosa, Marta N. Shahbazi

**Affiliations:** MRC Laboratory of Molecular Biology, Cambridge, CB2 0QH, UK.

## Abstract

Primordial germ cells (PGCs) are the precursors of gametes and the sole mechanism by which animals transmit genetic information across generations. In the mouse embryo, the transcriptional and epigenetic regulation of PGC specification has been extensively characterized. However, the initial event that triggers the soma-germline segregation remains poorly understood. Here, we uncover a critical role for the basement membrane in regulating germline entry. We show that PGCs arise in a region of the mouse embryo that lacks contact with the basement membrane, and the addition of exogenous extracellular matrix (ECM) inhibits both PGC and PGC-like cell (PGCLC) specification in mouse embryos and stem cell models, respectively. Mechanistically, we demonstrate that the engagement of β1 integrin with laminin blocks PGCLC specification by preventing the Wnt signaling–dependent down-regulation of the PGC transcriptional repressor, Otx2. In this way, the physical segregation of cells away from the basement membrane acts as a morphogenetic fate switch that controls the soma-germline bifurcation.

## Teaser

Extracellular matrix signaling via integrins acts as a morphogenetic checkpoint that restricts entry into the mouse germ line.

## INTRODUCTION

Primordial germ cells (PGCs) are the undifferentiated precursors of gametes and one of the earliest cell types to specify during embryonic development (*1*). In mice, PGCs are specified from embryonic epiblast cells in response to high levels of bone morphogenetic protein (Bmp) signaling on embryonic day 6.5 (E6.5), shortly after the embryo implants in the uterus (*2*–*4*). An anterior-posterior (AP) gradient of Bmp4 is established by the combined activity of two extra-embryonic tissues, the extra-embryonic ectoderm (ExE) and the visceral endoderm (VE). The ExE acts as a source of Bmp4, while a subpopulation of VE cells, the anterior VE, secretes Bmp inhibitors (*5*). Downstream of Bmp signaling, PGC induction requires Wnt activation (*6*) and down-regulation of the transcriptional repressor, Otx2 (*7*), which triggers the expression of canonical PGC transcription factors such as Blimp1, Prmd14, and Ap2γ (*8*–*10*). As a result, PGC specification is restricted to a cluster of approximately 40 founder cells in the proximal, posterior postimplantation epiblast. While proximal posterior epiblast cells are exposed to a common signaling environment, not all of them down-regulate Otx2 and become PGCs (*11*). For this reason, the trigger that causes the separation of cells in the embryo into soma and germ line is not known.

As PGCs become specified, posterior epiblast cells undergo a series of morphological changes in preparation for gastrulation, namely, changes in cell shape and cell-cell adhesion, loss of polarized epithelial organization, as well as remodeling of the underlying basement membrane (*5*, *12*–*14*). This led us to explore whether some of these morphogenetic changes might modulate gene expression and PGC specification, to help ensure that the right number of PGCs become specified at the right place and time. In exploring this question, we uncovered a previously unknown morphogenetic checkpoint that gates germline entry in the mouse embryo.

## RESULTS

### PGCs detach from the basement membrane

**Fig. 1.**
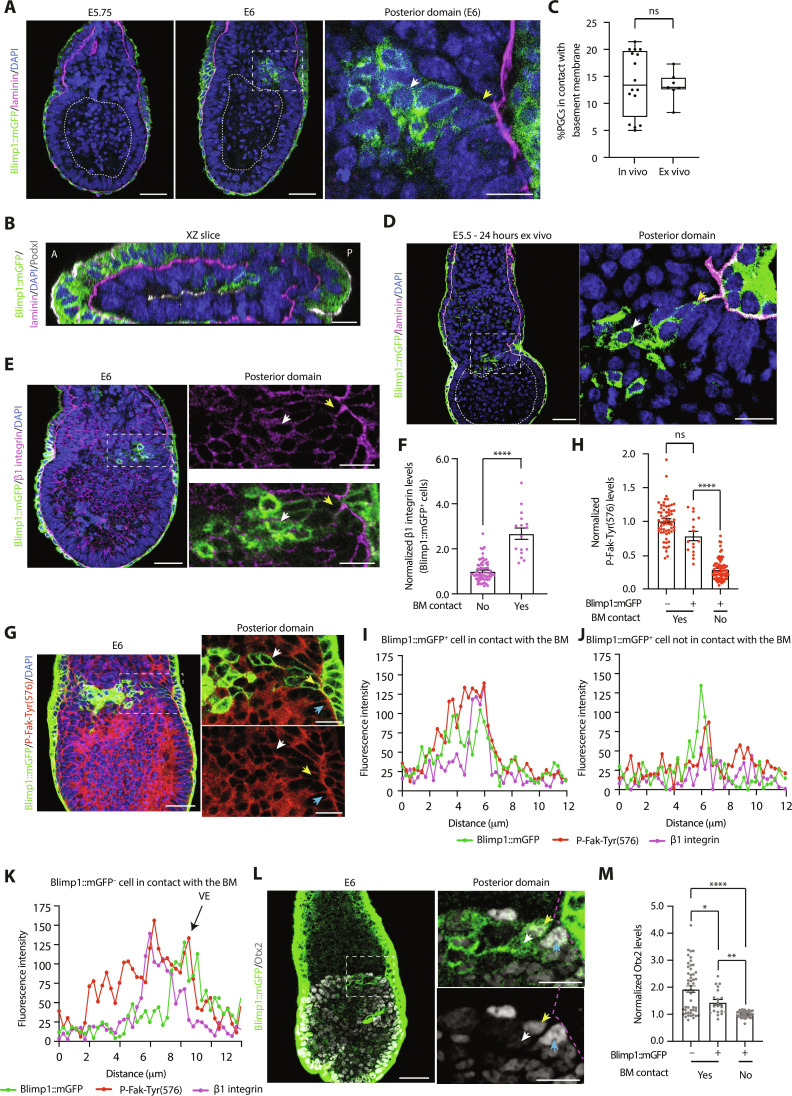
PGCs lose contact with the basement membrane. (**A**) Immunostaining of embryos developing in vivo. (**B**) XZ slice through an embryo. (**C**) Percentage of Blimp1::mGFP^+^ cells in contact with the basement membrane (BM) in embryos from (A) and (D). Data are shown as a box-and-whisker plot. *n* = 16 embryos, six independent experiments (in vivo). *n* = 7 embryos, three independent experiments (ex vivo). Welch’s *t* test. ns, nonsignificant. (**D** and **E**) Immunostaining of embryos cultured ex vivo (D) and developing in vivo (E). (**F**) β1 integrin levels in embryos from (E). Data are shown as mean ± SEM. Each point represents an individual cell. *n* = 67 (no) and *n* = 16 (yes) cells. Six embryos. Mann-Whitney *U* test. ****P < 0.0001. (**G**) Immunostaining of embryos developing in vivo. (**H**) P-Fak-Tyr(576) levels in embryos from (G). Data are shown as mean ± SEM. Each point represents an individual cell. *n* = 63 (yes, Blimp1::mGFP^−^), *n* = 16 (yes, Blimp1::mGFP^+^), and *n* = 71 (no, Blimp1::mGFP^+^) cells. Five embryos, two independent experiments. Kruskal-Wallis test. ****P < 0.0001. (**I** to **K**) Line profiles perpendicular to the membrane of cells marked with arrows in (G). (**L**) Immunostaining of embryos developing in vivo. Dashed line denotes the BM. (**M**) Otx2 levels in embryos from (L). Data are shown as mean ± SEM. Each point represents an individual cell. *n* = 51 (yes, Blimp1::mGFP^−^), *n* = 20 (yes, Blimp1::mGFP^+^), and *n* = 36 (no, Blimp1::mGFP^+^) cells. Six embryos, two independent experiments. One-way analysis of variance (ANOVA) with Welch’s correction. *P = 0.0102, **P = 0.0011, and ****P < 0.0001. For all panels, dashed lines denote the proamniotic cavity. Scale bars, 50 and 20 μm (magnified regions and XZ slice). Arrows indicate Blimp1::mGFP^−^ BM contact (blue), Blimp1::mGFP^+^ BM contact (yellow), and Blimp1::mGFP^+^ no BM contact (white).

We first sought to characterize the spatial context in which PGCs arise in the posterior epiblast using Blimp1::mGFP reporter embryos, on the basis that Blimp1^+^ cells represent the first lineage-restricted PGC precursors (*15*). Immunostainings of fixed embryos revealed that approximately 86% of Blimp1::mGFP^+^ PGCs, which begin to appear at E6, are localized in a region of the posterior epiblast that is not directly in contact with the laminin-rich basement membrane that surrounds the epiblast (Fig. 1, A to C, and movie S1). Approximately 14% of putative PGCs maintained contact with laminin at this stage of development (Fig. 1C), although there was variability from embryo to embryo. This finding was consistent for both embryos grown in vivo, as well as E5.5 embryos recovered from the uterus and cultured ex vivo for 24 hours (Fig. 1, C and D). Next, we analyzed whether there were gene expression differences in Blimp1::mGFP^+^ cells depending on whether they were in contact with the basement membrane. β1 integrin is the major laminin receptor (*16*), and Blimp1::mGFP^+^ cells that lacked basement membrane contact expressed lower levels of β1 integrin on their surface (Fig. 1, E and F). In agreement with the reduced β1 integrin levels, cells that lost contact with the extracellular matrix (ECM) also displayed lower levels of phosphorylated focal adhesion kinase (Fak) on tyrosine residue 576, P-Fak-Tyr(576) (Fig. 1, G to K), a readout of β1 integrin signaling (*17*). Moreover, Blimp1::mGFP^+^ cells in contact with the basement membrane expressed significantly higher levels of the PGC transcriptional repressor, Otx2 (Fig. 1, L and M). The same spatial expression pattern of Otx2 was observed in E5.5 embryos cultured ex vivo for 24 hours (fig. S1, A and B). Therefore, epiblast cells that enter the germ line have reduced levels of β1 integrin and reduced interactions with the underlying basement membrane and lower levels of Otx2.

To determine whether the loss of integrin-dependent basement membrane interactions might play a causal role during PGC specification, we devised a series of experiments to manipulate the basement membrane in ex vivo–cultured embryos. First, we explored whether we could mimic basement membrane signaling in embryos cultured ex vivo. To this end, we dissolved growth factor–reduced Matrigel, a rich source of ECM proteins, at a dilution of 5% in the embryo culture medium, recovered E6 to E6.5 embryos, and removed the VE layer to allow epiblast cells to interact with the exogenous ECM proteins (fig. S1C). We then cultured the epiblast-extraembryonic ectoderm fragments for 48 hours ex vivo in the presence or absence of Matrigel. In a subset of embryos, the addition of Matrigel was found to completely block PGC specification, while embryos that went on to develop PGCs in the presence of Matrigel appeared indistinguishable from controls (fig. S1, D to F). This experiment suggested that Matrigel may compromise PGC specification but not maintenance. Therefore, we next assessed whether Matrigel addition would alter PGC numbers at a later developmental stage. Accordingly, we recovered E7.5 embryos, removed the VE layer, and cultured the epiblast-ExE fragments for 24 hours ex vivo in the presence or absence of Matrigel. In this case, Matrigel addition did not affect PGC numbers (fig. S1, G and H). Globally, our experiments demonstrate that basement membrane signaling inhibits germline entry in a subset of embryos cultured ex vivo, but it does not affect PGC maintenance.

A recent report has documented the existence of basement membrane perforations in the posterior epiblast, before the onset of gastrulation (*12*). Therefore, to test whether the degradation of the basement membrane might facilitate PGC specification, we recovered E5.5 embryos, treated them with matrix metalloproteinase (MMP) inhibitors (*12*), and cultured them for 24 hours ex vivo. We chose E5.5 embryos for this experiment, since at this stage neither localized basement membrane perforations are observed (*12*), nor PGCs are specified (Fig. 1A). As previously shown, MMP inhibition led to a decrease in epiblast size (*12*) and aspect ratio (fig. S1I). Moreover, we noted that Nanog was expressed throughout the epiblast, instead of being restricted to the proximal-posterior region, indicating an altered formation of the AP axis (fig. S1J). Despite these defects, PGCs were specified at similar numbers to controls (fig. S1, K and L). Furthermore, the percentage of Blimp1::mGFP^+^ cells in contact with the basement membrane was unchanged between the experimental and control groups (fig. S1M), indicating that basement membrane remodeling is not required for the detachment and specification of putative PGCs. Globally, our experiments show that during PGC specification, epiblast cells decrease their interactions with the basement membrane and down-regulate Otx2.

### ECM prevents PGCLC specification

**Fig. 2.**
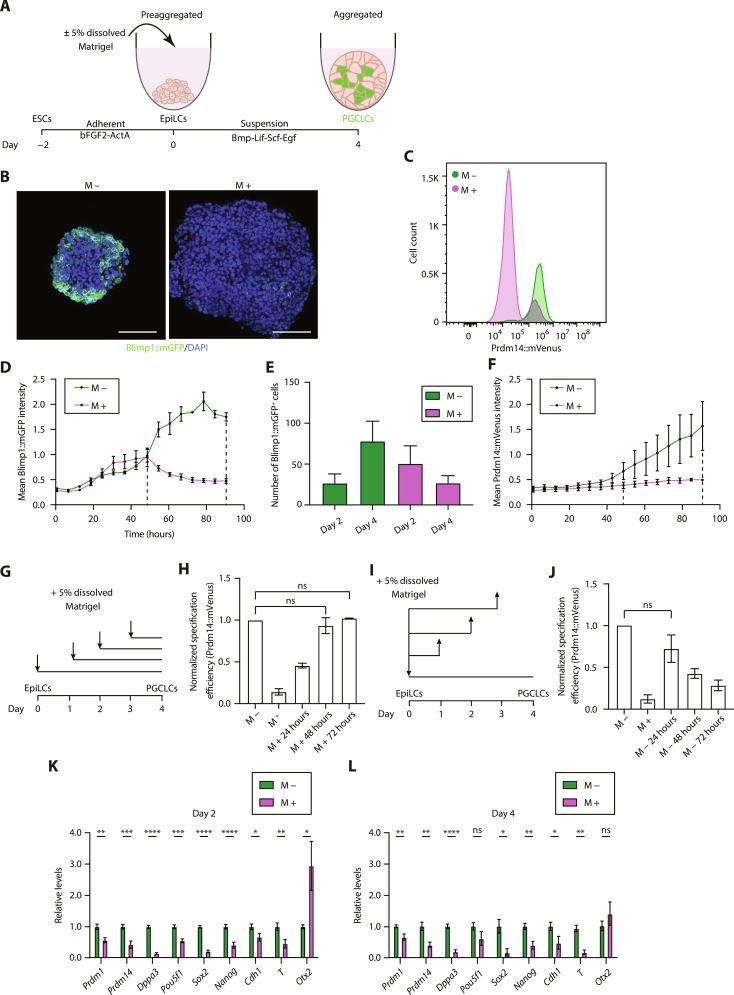
Exogenous ECM inhibits PGCLC specification. (**A**) Schematic of in vitro PGCLC differentiation protocol. (**B**) Immunostaining of EBs cultured without (M −) or with (M +) Matrigel. Scale bars, 100 μm. (**C**) Flow cytometry histogram showing the fluorescence intensity distribution of single cells from EBs. Four independent experiments. (**D**) Live-cell imaging analysis of EBs. Black dashed lines denote time points at days 2 and 4. Data are shown as mean ± SEM. *n* = 4 samples. Four independent experiments. (**E**) Bar graph showing the number of Blimp1^+^ cells (analyzed by flow cytometry) in EBs. Data are shown as mean ± SEM. *n* = 5 samples. 5 independent experiments. (**F**) Live-cell imaging analysis of EBs. Black dashed lines denote time points at days 2 and 4. Data are shown as mean ± SEM. *n* = 2 samples. Two independent experiments. (**G**) Schematic of the Matrigel addition time course experiment. (**H**) PGCLC specification efficiency (analyzed by flow cytometry) in EBs cultured as shown in (G). Data are shown as mean ± SEM. *n* = 2 samples. Two independent experiments. Kruskal-Wallis test. (**I**) Schematic of the Matrigel removal time course experiment. (**J**) PGCLC specification efficiency (analyzed by flow cytometry) in EBs cultured as shown in (I). Data are shown as mean ± SEM. *n* = 2 samples. Two independent experiments. Kruskal-Wallis test. (**K** and **L**) Time course gene expression analysis [quantitative reverse transcription polymerase chain reaction (qRT-PCR)] of EBs cultured without (M −) or with (M +) Matrigel. Data are shown as mean ± SEM. *n* = 10 samples. Six independent experiments. Unpaired Student’s *t* test. 0.01 < *P < 0.05, 0.001 < **P < 0.01, 0.0001 < ***P < 0.001, and ****P < 0.0001.

To tease apart the mechanism by which basement membrane signaling might inhibit PGC specification, we turned to an established embryonic stem cell (ESC)–based model for PGC-like cell (PGCLC) specification (*18*). Starting from ESCs, we induced epiblast-like cells (EpiLCs), which are competent to form PGCLCs when cultured in suspension for 4 days as three-dimensional (3D) cell aggregates, or embryoid bodies (EBs), in a Bmp-rich medium. We then asked whether ECM signaling, induced by the addition of 5% dissolved Matrigel to the medium, would inhibit PGCLC specification in Blimp1::mGFP (*15*) and Prdm14::mVenus (*19*) reporter EpiLCs (Fig. 2A). Notably, in both cases, we found that Matrigel addition was sufficient to inhibit PGCLC induction, compared to control (Fig. 2, B and C, and fig. S2A). Similar results were obtained when using Geltrex as a source of ECM proteins (fig. S2B). In the course of these experiments, we noted that EBs cultured with Matrigel grew significantly larger than controls (fig. S2C), presumably due to the known prosurvival role of the ECM (*20*, *21*). To ensure that PGCLC specification was not being confounded by these changes in size, we cultured EBs using variable numbers of seeded cells and showed that the number of cells in the EBs did not affect PGCLC specification in either the control or experimental groups (fig. S2D).

To better understand the effects of ECM signaling on the dynamics of PGCLC specification, we used a combination of live-cell imaging and time course immunostaining. Live-cell imaging of Blimp1::mGFP and Prdm14::mVenus EBs allowed us to quantify the mean fluorescence intensities at regular, 6-hour intervals (fig. S2E). This analysis revealed that, in the presence of Matrigel, Blimp1 up-regulation proceeded largely unperturbed within the first 36 to 48 hours of induction (Fig. 2D). Beyond 48 hours, there was a marked divergence in mean Blimp1::mGFP intensity between the control and Matrigel-treated groups, in which the Blimp1::mGFP signal underwent a pronounced decline by day 4 (Fig. 2D), in agreement with the immunofluorescence data (Fig. 2B). These results were validated by analyzing the total number of Blimp1::mGFP^+^ cells by flow cytometry at days 2 and 4. While in control conditions the number of Blimp1::mGFP^+^ cells increased from day 2 to day 4, in the presence of Matrigel Blimp1::mGFP^+^ cells decreased with time (Fig. 2E). Since Prdm14 is downstream of Blimp1, Prdm14::mVenus was slightly delayed in its onset and proceeded to increase in intensity under control conditions while completely failing to do so in the presence of ECM (Fig. 2F). These results suggest the presence of a critical, 48-hour period at the start of PGCLC induction that is sensitive to the presence of ECM proteins and during which a robust developmental program must be established to successfully induce PGCLCs.

To further explore this idea, we set up two time course experiments where we either added or removed Matrigel at intermediate time points during PGCLC induction and observed its effects on PGCLC specification. In the first, so-called Matrigel addition time course, we set up four conditions where we added Matrigel on either day 0, day 1, day 2, or day 3 (Fig. 2G). We were able to show that adding Matrigel as late as day 2 onward had no inhibitory effect on PGCLC specification (Fig. 2H). In the second, so-called Matrigel removal time course, we set up four conditions where we added Matrigel on day 0 and then proceeded to remove it on either day 1, day 2, or day 3 (Fig. 2I). Consistent with the findings of the Matrigel addition time course, we found that removing Matrigel as early as day 1 led to complete recovery of PGCLC specification, whereas Matrigel removal on or after day 2 led to decreasing PGCLC specification efficiencies (Fig. 2J). Together, these results support the notion that the inhibitory effect of ECM signaling on PGCLC specification only acts within the first 48 hours of induction.

Next, we sought to analyze the temporal changes in gene expression due to ECM signaling during PGCLC specification. As early as day 2, several canonical PGC marker genes, as well as several reacquired pluripotency genes, were significantly down-regulated in EBs cultured with Matrigel (Fig. 2K and fig. S2F). Notably, *Cdh1* (E-cadherin) expression, which mediates cell-cell adhesions during early PGC development in vivo and is required for their specification (*22*), was markedly decreased (Fig. 2K). Likewise, the mesodermal gene, *T* (Brachyury), a downstream effector of Wnt signaling that is transiently expressed and essential for activation of early germline determinants (*23*), only reached very low levels of expression in the presence of ECM (Fig. 2K). Conversely, *Otx2* was expressed at significantly higher levels in the presence of ECM signaling on day 2 (Fig. 2K). As expected, we observed a negative correlation between Brachyury and Otx2 in EBs analyzed by immunofluorescence at day 2, whereby cells that down-regulate Otx2 express high levels of Brachyury (fig. S2G). These global gene expression changes on day 2 led to a clear failure of PGCLC specification by day 4 (Fig. 2L and fig. S2H). Next, we explored whether Blimp1::mGFP^+^ cells display different gene expression profiles, depending on the presence or absence of Matrigel in the medium. To do this, we sorted the GFP^−^ and GFP^+^ populations from Blimp1::mGFP reporter EBs at days 2 and 4 and analyzed the expression of PGC genes. This revealed that GFP^+^ cells display the expected changes in gene expression, namely, the down-regulation of Otx2, up-regulation of T, and up-regulation of canonical PGC markers, regardless of whether Matrigel is present (fig. S2, I and J). Therefore, the few PGCLCs that appear in the presence of Matrigel do not display major alterations in gene expression. Put together, these data demonstrate that ECM signaling abrogates the initiation of PGCLC specification rather than its maintenance.

### ECM inhibits Wnt-mediated Otx2 down-regulation

**Fig. 3.**
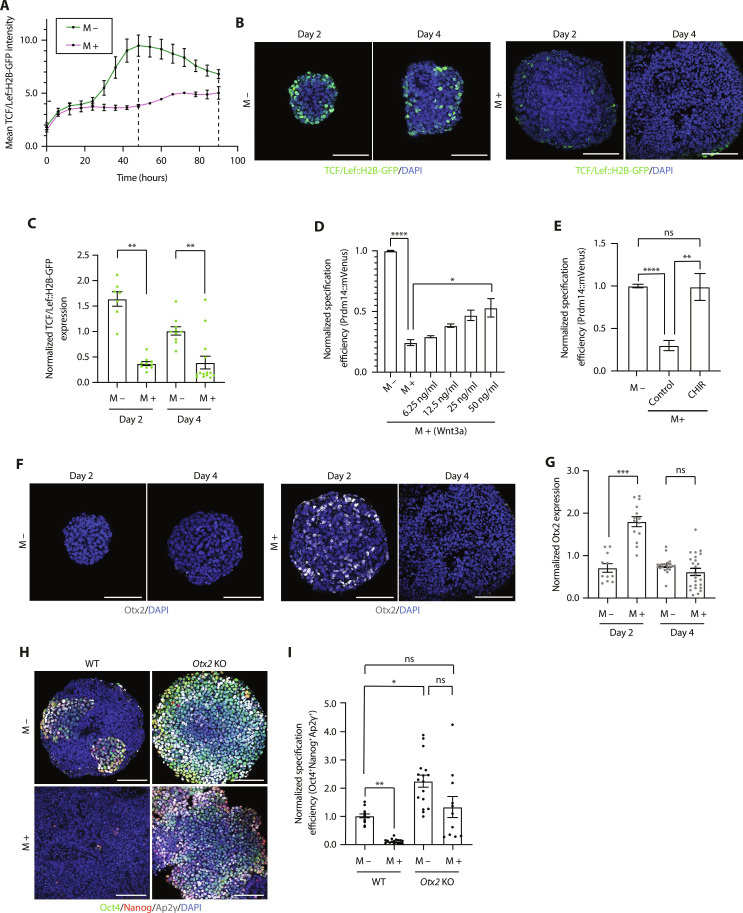
ECM inhibits Wnt signaling and Otx2 down-regulation. (**A**) Live-cell imaging analysis of EBs. Black dashed lines denote days 2 and 4. Data are shown as mean ± SEM. *n* = 3 samples. Three independent experiments. (**B**) Time course immunostaining of EBs. (**C**) Wnt activity in EBs from (B). Data are shown as mean ± SEM. Each point represents an EB. *n* = 7 (M − day 2), *n* = 10 (M + day 2), *n* = 10 (M − day 4), and *n* = 14 (M + day 4) EBs. Three independent experiments. Kruskal-Wallis test. **P = 0.0029 (day 2) and **P = 0.0065 (day 4). (**D** and **E**) PGCLC specification efficiency (analyzed by flow cytometry). Data are shown as mean ± SEM. For (D), *n* = 7 samples. Four independent experiments. Kruskal-Wallis test. *P = 0.0139 and ****P < 0.0001. For (E), *n* = 12 samples. Seven independent experiments. Kruskal-Wallis test. **P = 0.0074 and ****P < 0.0001. CHIR, Wnt activator CHIR99021. (**F**) Time course immunostaining of EBs. (**G**) Otx2 expression in EBs from (F). Data are shown as mean ± SEM. Each point represents an EB. *n* = 11 (M − day 2), *n* = 14 (M + day 2), *n* = 20 (M − day 4), and *n* = 24 (M + day 4) EBs. Four independent experiments. Kruskal-Wallis test. ***P = 0.0006. (**H**) Immunostaining of EBs. (**I**) PGCLC specification efficiency in EBs from (H). Data are shown as mean ± SEM. Each point represents an EB. *n* = 16 (WT M −), *n* = 17 (WT M +), *n* = 17 (*Otx2* KO M −), and *n* = 11 (*Otx2* KO M +) EBs. Two independent experiments. Kruskal-Wallis test. *P = 0.0362 and **P = 0.0011. Scale bars, 100 μm.

We next wanted to unravel how ECM signaling may be affecting critical, downstream cell signaling pathways during PGCLC induction. Canonical Wnt signaling acts downstream of Bmp signaling to down-regulate Otx2 expression, activate PGC transcription factors, and establish the PGC fate (*7*, *23*). Using a Bmp reporter line, IBRE4::CFP (*24*), and a Wnt reporter line, TCF/Lef::H2B-GFP (*25*), we analyzed the dynamics of these signaling pathways in the presence of ECM. Time course immunostaining of IBRE4::CFP EBs revealed that Bmp signaling activity was unperturbed across time and showed no differences between the experimental and control groups (fig. S3, A and B). On the other hand, live-cell imaging of TCF/Lef::H2B-GFP EBs revealed divergent Wnt signaling activity between the experimental and control groups beyond the first 24 hours of PGCLC induction, culminating in a distinct peak of Wnt signaling activity after 48 hours that was completely absent in the Matrigel-treated group (Fig. 3A). This peak of Wnt signaling activity was only transiently maintained, diminishing beyond the first 48 hours, thereby corroborating the notion of there being a narrow window of competence. The same trends were observed by time course immunostaining of TCF/Lef::H2B-GFP EBs (Fig. 3, B and C), showing significant down-regulation of Wnt signaling activity in the Matrigel-treated condition as early as day 2 (Fig. 3C), which was also reflected in the significant down-regulation of the downstream Wnt effector, Brachyury (fig. S3, C and D). We conclude from these experiments that ECM signaling inhibits Wnt signaling activity, downstream of Bmp signaling, within the first 48 hours of induction.

To functionally test whether the observed PGCLC specification defect was a direct consequence of the failure to activate canonical Wnt signaling, we tested the effects of exogenous Wnt activation on PGCLC specification in the presence of ECM, by two independent methods. First, we added varying concentrations of recombinant Wnt3a to activate the Wnt signaling pathway from its most upstream point (*23*). Under these conditions, we observed a partial rescue in the presence of ECM, with a clear dose-response (Fig. 3D). At 50 ng/ml, the PGCLC specification efficiency was significantly higher than the test condition but still failed to reach control levels (Fig. 3D). The ability of Wnt3a to partially rescue the effects of ECM implies that ECM acts to inhibit Wnt signaling. This led us to target glycogen synthase kinase 3 (GSK3), a known component of the intracellular β-catenin destruction complex and inhibitor of Wnt signaling (*26*), using the GSK3 inhibitor, CHIR99021. In the presence of both ECM and this exogenous Wnt activator, we observed full recovery of PGCLC specification to control levels on day 4 (Fig. 3E), while EBs grown in the absence of ECM appeared unaffected by the exogenous Wnt activation (fig. S3E). These results further demonstrate that the ECM blocks PGCLC specification by inhibiting Wnt signaling.

Given that Wnt signaling is known to repress Otx2 (*7*) and Otx2 mRNA levels are sustained in the presence of Matrigel (Fig. 2K), we next questioned whether Otx2 inhibits PGCLC specification downstream of ECM-mediated Wnt inhibition. First, we validated the increased protein levels of Otx2, specifically on day 2 of PGCLC induction in the presence of Matrigel (Fig. 3, F and G). Further to this, we used an *Otx2* knockout (KO) cell line. In the absence of Otx2, PGCLC specification efficiency was increased as previously described (*7*). In the presence of ECM, the loss of Otx2 restored PGCLC specification efficiency to control levels (Fig. 3, H and I). Together, these data strongly support the role of Wnt signaling in down-regulating Otx2. Thus, ECM blocks PGCLC specification by inhibiting Wnt signaling and subsequent Otx2 down-regulation.

### β1 integrin blocks PGCLC specification

Having established the role of ECM in inhibiting PGCLC specification by inhibiting Wnt signaling and downstream Otx2 down-regulation, we next sought to establish a causal link between ECM and Wnt signaling. We noticed that EBs grown with exogenous ECM consistently showed loss of adherens junctions through significantly down-regulated E-cadherin expression (fig. S4, A and B). PGCs in vivo actively up-regulate epithelial genes such as E-cadherin (*27*), which plays a critical role in the homotypic clustering of PGC precursors (*22*, *28*). In other systems, E-cadherin has also been shown to play a role in potentiating Wnt-dependent stem cell differentiation, through the timely sequestration of β-catenin to protect it from cytoplasmic degradation (*29*). These observations led us to test the idea that ECM may compromise E-cadherin–mediated cell-cell adhesions, leading to the observed PGCLC specification defect. To test this, we overexpressed E-cadherin using a doxycycline (Dox)–inducible system. As a control, we first validated that increasing the concentration of Dox induced increasing levels of GFP-tagged E-cadherin (fig. S4, A to C). At 10 μg/ml Dox, we were able to restore E-cadherin expression to control levels (fig. S4B). Unexpectedly, we found that while ectopic overexpression of E-cadherin was sufficient to up-regulate AP2γ in the Matrigel condition, these AP2γ^+^ cells did not coexpress Nanog, and therefore the PGCLC levels were not restored (fig. S4, D to F). These findings led us to conclude that the restoration of E-cadherin expression is not sufficient to rescue the specification of PGCLCs in the presence of ECM.

**Fig. 4.**
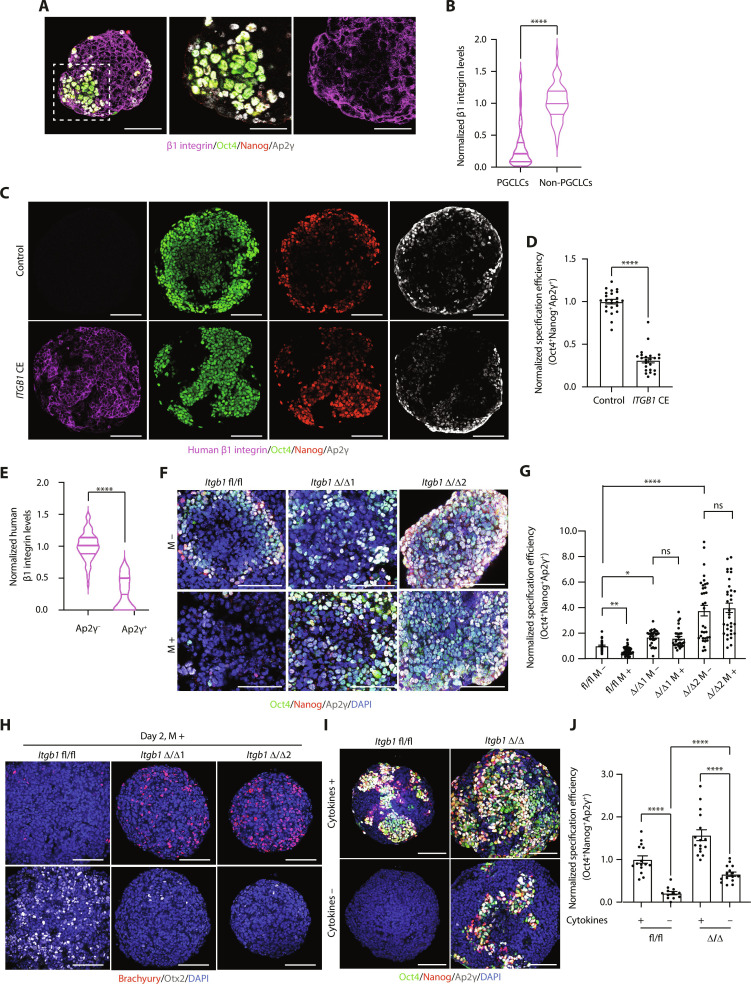
β1 integrin inhibits PGCLC specification. (**A**) Immunostaining of EBs. (**B**) Violin plots showing β1 integrin levels in EBs from (A). Data are shown as mean and interquartile range (IQR). *n* = 50 (PGCLCs) and *n* = 50 (non-PGCLCs) EBs. Four independent experiments. Mann-Whitney *U* test. ****P < 0.0001. (**C**) Immunostaining of control and *ITGB1* constitutively expressing (CE) EBs. (**D**) PGCLC specification efficiency in EBs from (C). Data are shown as mean ± SEM. Each point represents an EB. *n* = 23 (control) and *n* = 23 (*ITGB1* CE) EBs. Two independent experiments. Mann-Whitney *U* test. ****P < 0.0001. (**E**) Violin plots showing human β1 integrin levels in EBs from (C). Data are shown as mean and IQR. *n* = 63 (Ap2γ^+^) and *n* = 63 (Ap2γ^−^) EBs. Four independent experiments. Mann-Whitney *U* test. ****P < 0.0001. (**F**) Immunostaining of control (fl/fl) and *Itgb1* KO (Δ/Δ1, Δ/Δ2) EBs. (**G**) PGCLC specification efficiency in EBs from (F). Data are shown as mean ± SEM. Each point represents an EB. *n* = 42 (fl/fl M −), *n* = 52 (fl/fl M +), *n* = 30 (Δ/Δ1 M −), *n* = 31 (Δ/Δ1 M +), *n* = 34 (Δ/Δ2 M −), and *n* = 33 (Δ/Δ2 M +) EBs. Six independent experiments. Kruskal-Wallis test. *P = 0.0334, **P = 0.0054, and ****P < 0.0001. (**H** and **I**) Immunostaining of EBs. (**J**) PGCLC specification efficiency in EBs from (I). Data are shown as mean ± SEM. Each point represents an EB. *n* = 15 (fl/fl, cytokines +), *n* = 12 (fl/fl, cytokines −), *n* = 16 (Δ/Δ, cytokines +), and *n* = 15 (Δ/Δ, cytokines −) EBs. Two independent experiments. One-way ANOVA with Welch’s correction. ****P < 0.0001. Scale bars, 100 and 50 μm (magnified regions).

Next, we decided to determine whether integrins, being the main receptors of ECM proteins, mediate the inhibitory effect of ECM on PGCLC specification. Specifically, we focused our attention on β1 integrin, as it is the major laminin receptor (*16*). Since β1 integrin is down-regulated upon germline entry in embryos, we first analyzed whether this was also happening in PGCLCs. Analysis of β1 integrin levels in EBs showed that PGCLCs express significantly lower levels of β1 integrin compared to non-PGCLCs (Fig. 4, A and B, and fig. S5A). Therefore, we first explored the consequences of β1 integrin constitutive expression in EBs, where we saw that forced up-regulation of β1 integrin levels led to a significant decrease in PGC numbers (Fig. 4, C and D). We observed that while Oct4 and Nanog broadly colocalized in β1 integrin constitutively-expressing cells, these double-positive cells did not express AP2γ (Fig. 4E). AP2γ was preferentially expressed in regions that did not constitutively express β1 integrin (Fig. 4, C and E). These results show that while E-cadherin up-regulation triggers AP2γ expression (fig. S4, D and E), β1 integrin up-regulation inhibits AP2γ expression.

To determine the functional consequences of β1 integrin loss, we generated *Itgb1* KO ESCs by Cre-Lox recombination of a floxed *Itgb1* allele, isolated two separate clones (*Itgb1* Δ/Δ1 and *Itgb1* Δ/Δ2), and used *Itgb1* fl/fl ESCs as a control (fig. S5B). While we observed the same PGCLC specification defect in *Itgb1* fl/fl ESCs in the presence of Matrigel (Fig. 4F), we found that both *Itgb1* KO clones did not respond to the inhibitory effects of ECM signaling (Fig. 4, F and G). Both *Itgb1* KO clones were considerably more permissive to PGCLC induction, yielding significantly higher PGCLC specification efficiencies compared to *Itgb1* fl/fl controls (Fig. 4, F and G). Globally, these data show that β1 integrin mediates the inhibitory effect of ECM signaling on PGCLC specification, as well as pointing to a much broader role of β1 integrin in modulating the responsiveness of PGC-competent cells to inductive PGC fate signaling.

We next sought to look at the downstream effects of *Itgb1*, specifically as they pertained to Wnt signaling and Otx2 levels. We cultured *Itgb1* fl/fl control and *Itgb1* KO EBs with 5% dissolved Matrigel and analyzed the expression of the downstream Wnt effector, Brachyury, and Otx2 on day 2 of PGCLC induction. This revealed that Brachyury levels were up-regulated, while Otx2 was down-regulated in the *Itgb1* KO EBs on day 2 compared to control EBs (Fig. 4H and fig. S5, C and D). These results demonstrate that activation of β1 integrin upon ECM binding restricts germline entry by blocking Wnt signaling.

Given that loss of Otx2 is sufficient to trigger germline entry in the absence of exogenous cytokines (*7*), we next analyzed whether the absence of β1 integrin was similarly permissive for germline entry. As expected, control cells failed to form PGCLCs in the absence of Bmp, leukemia inhibitory factor (LIF), epidermal growth factor (EGF), and stem cell factor (SCF) (Fig. 4, I and J). However, β1 integrin null cells showed a significant increase in PGCLC numbers compared to control cells (Fig. 4, I and J), although these numbers were nonetheless significantly lower than those observed in the presence of cytokines (Fig. 4, I and J). We therefore conclude that loss of β1 integrin is needed for PGCLC specification, but it is only partially sufficient to allow germline entry in the absence of Bmp stimulation.

### Laminin signaling blocks PGCLC specification

**Fig. 5.**
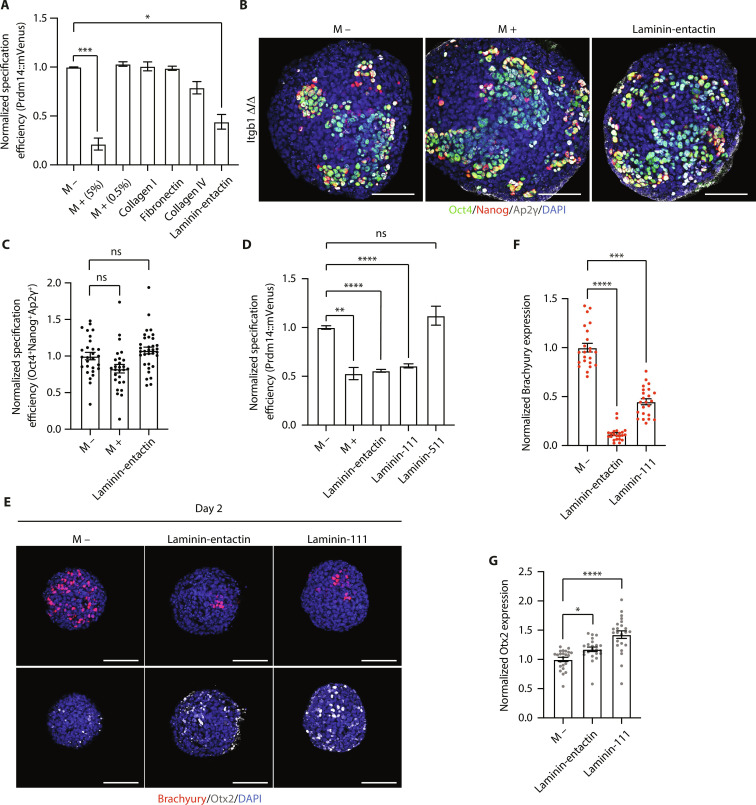
Laminin-111 inhibits PGCLC specification. (**A**) PGCLC specification efficiency (analyzed by flow cytometry) in EBs cultured without (M −) or with (M +) Matrigel or other ECM components. Data are shown as mean ± SEM. *n* = 7 (M −) and *n* = 6 (M + 5%) samples. Four independent experiments. *n* = 2 (M + 0.5%) samples. Two independent experiments. *n* = 5 (collagen I and fibronectin) and *n* = 4 (collagen IV and laminin-entactin) samples. Four independent experiments. Kruskal-Wallis test. ***P = 0.0007 and *P = 0.047. (**B**) Immunostaining of *Itgb1* KO (Δ/Δ) EBs. (**C**) PGCLC specification efficiency in EBs from (B). Data are shown as mean ± SEM. *n* = 28 (M −), *n* = 27 (M +), and *n* = 34 (laminin-entactin) EBs. Three independent experiments. Kruskal-Wallis test. (**D**) PGCLC specification efficiency (analyzed by flow cytometry). Data are shown as mean ± SEM. *n* = 7 (M −), *n* = 7 (M +), *n* = 5 (laminin-entactin), *n* = 7 (laminin-111), and *n* = 5 (laminin-511) samples. Three independent experiments. One-way ANOVA with Welch’s correction. **P = 0.0019 and ****P < 0.0001. (**E**) Immunostaining of EBs. (**F**) Brachyury expression in EBs from (E). Data are shown as mean ± SEM. Each point represents an EB. *n* = 23 (M −), *n* = 22 (laminin-entactin), and *n* = 23 (laminin-111) EBs. Two independent experiments. Kruskal-Wallis test. ***P = 0.0003 and ****P < 0.0001. (**G**) Otx2 expression in EBs from (E). Data are shown as mean ± SEM. Each point represents an EB. *n* = 23 (M −), *n* = 22 (laminin-entactin), and *n* = 23 (laminin-111) EBs. Two independent experiments. Kruskal-Wallis test. *P = 0.0343 and ****P < 0.0001. Scale bars, 100 μm.

Since Matrigel is a highly heterogeneous basement membrane preparation, and β1 integrin is activated by a variety of ECM proteins (*16*), we next wanted to identify the specific Matrigel component that activates β1 integrin to inhibit germline entry. On the basis of information about the protein composition of Matrigel (*30*), we individually tested each component, at its respective concentration, to see its effect on PGCLC specification. Laminin-entactin was the only ECM component that was sufficient, in isolation, to significantly inhibit PGCLC specification (Fig. 5A). *Itgb1* KO ESCs were also insensitive to the presence of laminin-entactin (Fig. 5, B and C). Since laminin is considerably more abundant in Matrigel than entactin (*30*) and is one of the most abundant ECM proteins in the basement membrane in vivo (*12*, *31*), we next explored which specific laminin subunit inhibits PGCLC specification. Notably, while addition of laminin-511 to the medium did not affect PGCLC specification, addition of laminin-111 was sufficient to fully recapitulate the effects of Matrigel in terms of PGCLC numbers (Fig. 5D). Moreover, both laminin-entactin and laminin-111 prevented Otx2 down-regulation and inhibited Brachyury up-regulation (Fig. 5, E to G). Therefore, we conclude that the inhibitory effect on PGCLC specification is mediated by laminin-111.

### Src blocks PGC specification in vitro and ex vivo

**Fig. 6.**
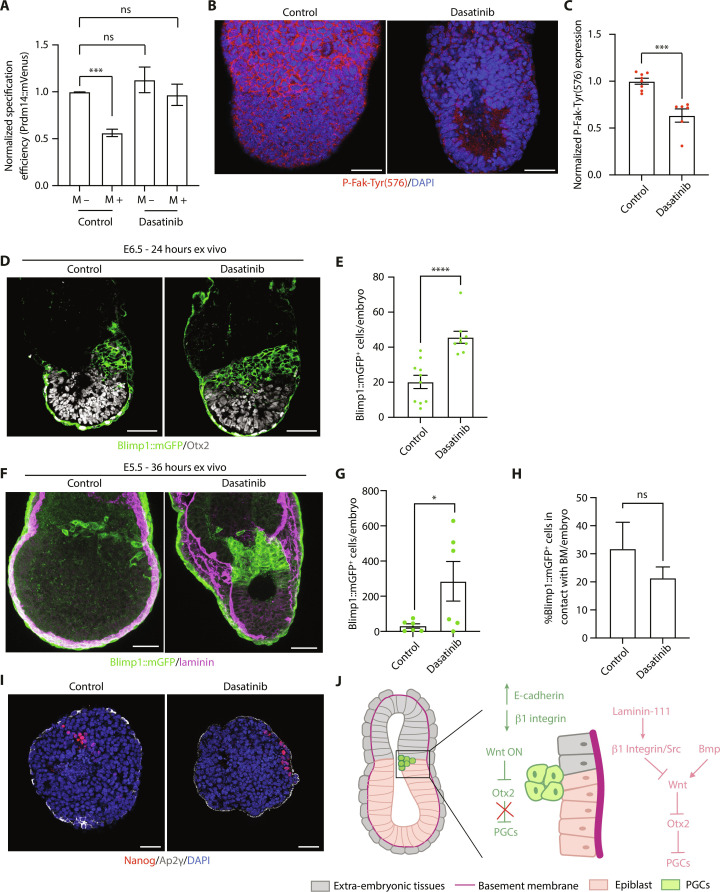
Src inhibits PGC specification. (**A**) PGCLC specification efficiency (analyzed by flow cytometry) in EBs cultured without (M −) or with (M +) Matrigel. Data are shown as mean ± SEM. *n* = 6 samples. Three independent experiments. One-way ANOVA Welch’s correction. ***P = 0.0005. (**B**) Immunostaining of E6.5 embryos, cultured ex vivo for 24 hours without (control) or with a Src inhibitor (Dasatinib). (**C**) P-Fak-Tyr(576) levels in embryos from (B). Data are shown as mean ± SEM. *n* = 8 (control) and *n* = 6 (Dasatinib) embryos. Three independent experiments. Mann-Whitney *U* test. ***P = 0.0007. (**D**) Immunostaining of E6.5 embryos, cultured ex vivo for 24 hours without (control) or with a Src inhibitor (Dasatinib). (**E**) Number of PGCs in embryos from (D). Data are shown as mean ± SEM. Each point represents an embryo. *n* = 10 (control) and *n* = 9 (Dasatinib) embryos. Three independent experiments. Mann-Whitney *U* test. ****P < 0.0001. (**F**) Immunostaining of E5.5 embryos, cultured ex vivo for 36 hours without (control) or with a Src inhibitor (Dasatinib). (**G**) Number of PGCs in embryos from (F). Data are shown as mean ± SEM. Each point represents an embryo. *n* = 6 (control) and *n* = 6 (Dasatinib) embryos. Three independent experiments. Unpaired Student’s *t* test. *P = 0.0486. (**H**) Percentage of Blimp1::mGFP^+^ cells in contact with the BM in embryos from (F). Data are shown as mean ± SEM. *n* = 6 (control) and *n* = 5 (Dasatinib) embryos. Three independent experiments. Unpaired Student’s *t* test. (**I**) Epiblast-VE fragments from E6.5 embryos, cultured ex vivo for 48 hours without (control) or with a Src inhibitor (Dasatinib). (**J**) Proposed model. ON means activated. Scale bars, 50 μm.

β1 integrin transduces extracellular signals via several intracellular kinases, including proto-oncogene tyrosine-protein kinase Src (Src) and integrin-linked kinase (Ilk), both with reported roles in the regulation of Wnt signaling (*29*, *32*, *33*). Therefore, we tested whether Src and Ilk block germline entry, similarly to β1 integrin, using a pharmacological approach. The use of a selective Ilk inhibitor, CPD-22 (*34*), did not affect PGCLC specification (fig. S5E). On the other hand, treatment with the Src inhibitor, Dasatinib (*35*), led to a full rescue of PGCLC numbers in the presence of Matrigel (Fig. 6A). To validate whether Src inhibition also promotes germline entry in the mouse embryo, we cultured E6.5 embryos ex vivo for 24 hours in the presence of Dasatinib. As a control for the experiment, we first validated that Dasatinib treatment decreased the levels of P-Fak-Tyr(576) (Fig. 6, B and C), a phosphorylation mediated by Src (*17*). In agreement with our in vitro results, Dasatinib treatment led to a significant increase in the number of PGCs (Fig. 6, D and E). At this stage, the posterior basement membrane was already broken, and therefore none of the PGCs were in contact with basement membrane proteins (fig. S5F). To analyze embryos at an earlier time point, we next recovered E5.5 embryos and cultured them ex vivo for 36 hours in the presence of Dasatinib. In agreement with our previous results, Dasatinib treatment from E5.5 also increased the number of PGCs (Fig. 6, F and G). In Dasatinib-treated embryos, PGCs extended toward the anterior and proximal regions of the embryo, and we did not observe any differences in basement membrane contact between control and Dasatinib-treated embryos (Fig. 6H). Lastly, we explored whether Dasatinib was sufficient to trigger germline entry in the absence of the ExE. To this end, we surgically removed the ExE from E6.5 embryos and cultured the VE-epiblast fragments for 48 hours. This experiment revealed that in the absence of ExE cells, Dasatinib treatment is not sufficient to trigger germline entry (Fig. 6I and fig. S5G). Similar results were obtained when isolated epiblasts (devoid of both ExE and VE) were cultured ex vivo in the presence of Dasatinib (fig. S5, H and I). Therefore, inhibition of Src is not sufficient to specify PGCs. Together, our data show that activation of β1 integrin/Src restricts pluripotent cell entry into the germ line.

## DISCUSSION

Here, we have shown that laminin signaling through β1 integrin receptors on germline-competent epiblast cells inhibits Wnt signaling and prevents Otx2 down-regulation, blocking PGC specification (Fig. 6J). This mechanism places both spatial and temporal constraints on PGC specification, acting as a morphogenetic fate switch. On the basis of our analyses of in vivo developing mouse embryos, we propose the following model: Bmp-mediated Wnt activation in posterior epiblast cells initiates the process of Otx2 down-regulation and Blimp1 activation. Detachment of cells from the basement membrane leads to a decrease of integrin signaling, which is needed to robustly activate the Wnt pathway and fully down-regulate Otx2. The mechanism that triggers the detachment from the basement membrane remains to be explored. Our experiments have shown that basement membrane breakdown is not necessary for this detachment, which could alternatively be regulated by changes in cell-cell adhesion in posterior epiblast cells (*36*), cell-ECM adhesion, or by increased tissue crowding (*37*, *38*) (see note added in proof). Likewise, the transcriptional mechanism that triggers β1 integrin down-regulation is unknown. Future experiments using time-lapse microscopy would be needed to characterize the dynamics of germline entry in the mouse embryo and determine the specific sequence of developmental events. It would be useful to generate Otx2 reporter and laminin reporter mouse models, which could be used in combination with the Blimp1 reporter.

While our results clearly demonstrate a role for β1 integrin during PGCLC specification in vitro, we have struggled to explore the function of β1 integrin in vivo. β1 integrin–deficient embryos are lethal at implantation (*39*, *40*), and while chimeras containing β1 integrin KO cells have been reported, only those that have a chimerism level below 25% are developmentally normal (*40*). These chimera studies have revealed that β1 integrin KO cells can enter the germ line (*41*), but whether increased PGC numbers are specified remains unexplored given the low level of chimerism. Alternative approaches are thus needed to explore the role of integrin signaling in vivo. To circumvent these limitations, here, we have pharmacologically targeted the downstream kinase Src and show that Src inhibition in embryos cultured ex vivo leads to a significant increase in the number of PGCs. Another possibility would be to focus on the α integrin subunits. We have seen that laminin-111 is the specific laminin subunit that blocks PGCLC specification. Since laminin-111 is frequently bound by α6β1 integrin (*42*), it would therefore be interesting to analyze germline entry in α6 integrin–deficient mice, which are not embryonically lethal (*43*). Moreover, while β1 integrin is necessary for PGC migration to the gonads, α6 integrin is not (*41*), and this would also represent a technical advantage when assessing both germline entry and subsequent development.

Mechanistically, our in vitro experiments have shown that β1 integrin inhibits PGCLC specification by preventing the Wnt-mediated down-regulation of the transcriptional repressor, Otx2. Otx2 expression is the main barrier for germline entry. Loss of Otx2 is sufficient to promote germline entry even in the absence of exogenous Bmp signaling and leads to a globally higher efficiency of PGCLC specification (*7*). Similarly, we have observed that in the absence of β1 integrin, there is a global increase in the number of PGCLCs, even when Bmp is not present. However, addition of PGC-inducing cytokines to the medium boosts PGCLC numbers, indicating that loss of β1 integrin signaling is necessary, but only partially sufficient, to trigger germline entry. In addition, we have seen that Src inhibition in ex vivo cultured embryos phenocopies the increased PGCLC numbers observed in β1 integrin KO cells, but it is not sufficient to trigger germline entry when ExE cells are not present. Further experiments are needed to unambiguously demonstrate that β1 integrin signaling blocks germline entry via Src activation. Moreover, how β1 integrin/Src affect Wnt activity is an important question that requires investigation. In cancer cells, Src inhibits Wnt signaling via phosphorylation of the Wnt coreceptor, Lrp6 (*44*), but whether this mechanism is active in the epiblast has not been explored. It is also unknown whether β1 integrin inhibits Wnt signaling in other developmental contexts, but it is tempting to speculate that basement membrane breakdown at the onset of gastrulation acts as a mechanism of boosting Wnt signaling in posterior epiblast cells.

Our results also show that ECM signaling triggers a down-regulation of the cell-cell adhesion protein, E-cadherin. This is an interesting finding given that E-cadherin is necessary for PGC specification (*22*), and we have seen that E-cadherin up-regulation leads to an increase in AP2γ, while β1 integrin overexpression decreases AP2γ levels. Our findings suggest that a switch from cell-ECM to cell-cell adhesions during PGC specification is critical. Beyond β1 integrin, other ECM receptors such as dystroglycans have also been shown to bind laminins (*45*), with dystroglycan and β1 integrin having overlapping functions during epithelialization in EBs (*46*). Whether dystroglycan also plays a role during PGC specification remains to be explored.

A recent publication has shown that the addition of Geltrex promotes human PGCLC specification (*47*). We have confirmed that both Matrigel and Geltrex have a similar inhibitory effect during mouse PGCLC specification. Whether this represents a species-specific difference remains to be further investigated. Experiments using different protocols and starting cell types for human PGCLC induction need to be considered, as it has been shown that the mechanism of human germline entry differs depending on the specific experimental approach that is used (*48*).

In summary, our work shows that mouse germline entry is restricted by β1 integrin–mediated inhibition of Wnt signaling. A tight coordination between morphogenesis, signaling, and cell fate would ensure that the right number of PGCs is specified at the right place and confers a robust developmental program during early mouse embryogenesis.

## MATERIALS AND METHODS

### Mouse work

All experiments involving mouse embryos were carried out in a UK Home Office–designated facility following national and international guidelines, regulated by the Animals (Scientific Procedures) Act 1986 following ethical review by the Laboratory of Molecular Biology (LMB) Animal Welfare and Ethical Review Body. Experiments were approved by the Home Office and carried out under the project license of M. Shahbazi (project license number PP4259105). For timed matings, the Blimp1::mGFP line (*15*) or CD1 wild-type (WT) mice were used. Embryos were recovered at E5.5 to E7.5 by manual dissection from the decidua, followed by careful removal of the Reichert’s membrane. The morning of the day the copulation plug was found was counted as E0.5.

### Embryo culture

Blimp1::mGFP or CD1 WT embryos were used for ex vivo culture. Dissection medium and ex utero embryo culture medium were preequilibrated at 37°C (21% O_2_ and 5% CO_2_), at least one hour before embryo recovery. Dissection medium comprised Dulbecco’s modified Eagle’s medium (DMEM; 11054020, Thermo Fisher Scientific) and 10% EmbryoMax fetal bovine serum (FBS) (ES-009-B, Merck Millipore).

For Matrigel treatment, E6 to 6.5 and E7 to 7.5 embryos were recovered, and the VE was removed as previously described (*6*). Briefly, embryos were incubated with 0.5% trypsin (15400054, Thermo Fisher Scientific) and 2.5% pancreatin (P3292, Sigma-Aldrich) dissolved in Eagle’s balanced salt solution (14155063, Thermo Fisher Scientific) for 7 min on ice. Embryos were then carefully pipetted with a narrow glass pipette matching the thickness of the epiblast to remove the VE. The remainder of the embryo (ExE and epiblast) was cultured ex vivo with or without 5% growth factor–reduced Matrigel (354230, Corning) dissolved in PGC medium. PGC medium comprised human bone morphogenetic protein 2 (500 ng/ml; hBMP-2) (M. Hyvonen lab, University of Cambridge), mouse leukemia inhibitory factor (50 ng/ml; mLIF) (Cambridge Stem Cell Institute), mouse stem cell factor (100 ng/ml; mSCF) (78064, STEMCELL Technologies), and mouse epidermal growth factor (50 ng/ml; mEGF) (PMG8043, Gibco) in GK15 base. GK15 base comprised Glasgow’s Minimal Essential Medium (GMEM; 11710035, Thermo Fisher Scientific), 15% KnockOut serum replacement (KSR; 10828028, Thermo Fisher Scientific), penicillin-streptomycin (15140122, Gibco), GlutaMAX (35050061, Thermo Fisher Scientific), MEM non-essential amino acids (11140035, Thermo Fisher Scientific), sodium pyruvate (11360070, Thermo Fisher Scientific), and 100 μM β-mercaptoethanol (31350010, Thermo Fisher Scientific).

For MMP inhibitor treatment, a previously described ex utero embryo culture medium was used (*49*). It comprised 25% DMEM (11054020, Thermo Fisher Scientific), 50% rat serum (provided by Charles River Laboratories), 25% human cord blood serum (provided by the Cambridge Blood and Stem Cell Biobank), penicillin-streptomycin (15140122, Gibco), GlutaMAX (35050061, Thermo Fisher Scientific), and Hepes buffer solution (15630056, Gibco). E5.5 embryos were recovered and cultured ex vivo for 24 hours with or without two MMP inhibitors: 20 μM prinomastat hydrochloride (PZ0198, Sigma-Aldrich) and 100 μM NSC 405020 (4902, Tocris Bioscience), as previously described (*12*). All embryos were cultured at 37°C, 21% O_2_, and 5% CO_2_.

For Src inhibitor treatment, E5.5 or E6.5 embryos were recovered and cultured ex vivo in GK15 base for 24 (E6.5) or 36 (E5.5) hours. The Src inhibitor, Dasatinib (*35*) (Cayman Chemical, 11498), was added at 1 μM. Where indicated, the ExE was manually removed using a finely pulled glass capillary, and the VE was removed, as described above.

### Mouse ESC culture

Mouse ESCs were cultured in Fc base medium supplemented with 2i/LIF (Fc2iL) on gelatin-coated plates. Fc2iL comprised 1 μM Mitogen-Activated Protein Kinase Kinase 1 (MEK) inhibitor (PD0325901, Cambridge Stem Cell Institute), 3 μM GSK3 inhibitor (CHIR99021, Cambridge Stem Cell Institute), and mLIF (10 ng/ml; Cambridge Stem Cell Institute). Fc base medium comprised DMEM (41966, Thermo Fisher Scientific), 15% FBS (10270-106, Gibco), penicillin-streptomycin (15140122, Gibco), GlutaMAX (35050061, Thermo Fisher Scientific), MEM non-essential amino acids (11140035, Thermo Fisher Scientific), sodium pyruvate (11360070, Thermo Fisher Scientific), and 100 μM β-mercaptoethanol (31350010, Thermo Fisher Scientific). Mouse ESCs were routinely passaged with trypsin-EDTA (produced in-house) at a ratio of 1:10. Fc base medium was used to neutralize the trypsin, and cells were centrifuged at 300*g* for 4 min. The following mouse ESC lines were used: Blimp1::mGFP (*15*) (generated in-house), Prdm14::mVenus (*19*) (courtesy of A. Meissner, Max Planck Institute for Molecular GeneticsGermany), *Itgb1* fl/fl (*50*) (courtesy of M. Żernicka-Goetz, University of Cambridge, UK), *Itgb1* Δ/Δ (generated in house), IBRE4::CFP (*24*) (courtesy of K. Zaret, University of Pennsylvania, USA), TCF/Lef::H2B-GFP (*25*) (generated in-house), E14 WT [courtesy of J. Nichols, Medical Research Council (MRC) Human Genetics Unit, UK], *Otx2* KO (*51*) (courtesy of C. Buecker, Max Perutz Laboratories, Austria), Dox-inducible E-cadherin–GFP (generated in-house), and ITGB1 constitutively expressing (generated in-house). The MycoAlert Mycoplasma Detection kit (LT07-118, Lonza) was used to routinely test all cell lines for mycoplasma. ESCs were cultured at 37°C, 21% O_2_, and 5% CO_2_.

### EpiLC induction

The first step of PGCLC induction involved the conversion of mouse ESCs into EpiLCs. Mouse ESCs were dissociated with trypsin-EDTA (produced in-house) and neutralized with Fc base medium. Cells were centrifuged at 300*g* for 4 min, washed with phosphate-buffered saline (PBS), centrifuged again, resuspended in EpiLC medium, and seeded on fibronectin-coated plates, at a density of 50 to 100,000 cells per well (12-well plate). Plates were coated by diluting fibronectin (1918-FN, R&D Systems) in PBS at 20 μg/ml and incubating for at least 1 hour at 37°C.

EpiLC medium comprised activin-A (20 ng/ml), basic fibroblast growth factor-2 (12 ng/ml) (M. Hyvonen lab, University of Cambridge), and 1% KSR (10828028, Thermo Fisher Scientific) in EpiSC base. EpiSC base comprised DMEM/F-12 (21331020, Thermo Fisher Scientific), 0.01% bovine serum albumin (BSA; A3311, Sigma-Aldrich), 1% (v/v) B27 (10889-038, Thermo Fisher Scientific), 0.5% (v/v) N2 (homemade), 100 μM β-mercaptoethanol (31350010, Thermo Fisher Scientific), penicillin-streptomycin (15140122, Gibco), MEM non-essential amino acids (11140035, Thermo Fisher Scientific), and GlutaMAX (35050061, Thermo Fisher Scientific). Homemade N2 supplement comprised DMEM-F/12 medium (21331-020, Thermo Fisher Scientific), 0.75% bovine albumin fraction V (15260037, Thermo Fisher Scientific), insulin (2.5 mg/ml; I9287, Sigma-Aldrich), apotransferrin (10 mg/ml; T1147, Sigma-Aldrich), progesterone (2 μg/ml; p8783, Sigma-Aldrich), sodium selenite (0.6 μg/ml; S5261, Sigma-Aldrich), and putrescine dihydrochloride (1.6 mg/ml; P5780, Sigma-Aldrich). Cells were cultured for 2 days in EpiLC medium.

### PGCLC induction

The second step of PGCLC induction involved the conversion of EpiLCs into PGCLCs. Cells were dissociated with TrypLE (12604021, Gibco) and neutralized with DMEM-F/12 (21331020, Thermo Fisher Scientific). Cells were centrifuged at 300*g* for 4 min, washed with PBS, and centrifuged again. Cells were resuspended in PGC medium and plated in 96-well, round-bottom, ultralow attachment microplates (Corning, 7007), at 2000 cells per well. Cells were initially seeded in 50 μl of PGC medium per well, and the plates were centrifuged at 120*g* for 5 min for the cells to sediment. After 1 to 2 hours of incubation at 37°C, and before a compacted aggregate was formed, an additional 50 μl of PGC medium was added, with or without dissolved growth factor–reduced Matrigel (354230, Corning), bringing the total volume per well to 100 μl. To achieve a final dissolved Matrigel dilution of 5%, 10% Matrigel was dissolved in 50 μl of cold PGC medium. Plates were then centrifuged again at 120*g* for 5 min.

Mouse PGC medium comprised hBMP-2 (500 ng/ml; M. Hyvonen lab, University of Cambridge), mLIF (50 ng/ml; Cambridge Stem Cell Institute), mSCF (100 ng/ml; 78064, STEMCELL Technologies), and mEGF (50 ng/ml; PMG8043, Gibco) in GK15 base. GK15 base comprised GMEM (11710035, Thermo Fisher Scientific), 15% KSR (10828028, Thermo Fisher Scientific), penicillin-streptomycin (15140122, Gibco), GlutaMAX (35050061, Thermo Fisher Scientific), MEM non-essential amino acids (11140035, Thermo Fisher Scientific), sodium pyruvate (11360070, Thermo Fisher Scientific), and 100 μM β-mercaptoethanol (31350010, Thermo Fisher Scientific). Cells were cultured for 4 days in PGC medium.

### ESC transfection

All vectors were transfected using Lipofectamine 3000 Transfection Reagent (Thermo Fisher Scientific), following the manufacturer’s instructions. To use the PiggyBac transposon system for chromosomal integration in ESCs, all inserts were cloned into PiggyBac-compatible vectors before transfection. For E-cadherin–GFP overexpression, Dox-inducible E-cadherin–GFP ESCs were made by transfecting WT E14 ESCs with the vector pZeo-TetO-ECAD-GFP previously described (*52*), along with a PBase vector and an rtTA3 vector containing a puromycin resistance cassette (courtesy of J. Silva, Guangzhou Laboratory). Transfected cells were expanded for at least 2 days, before antibiotic selection for an additional 7 days. Antibiotic selection was done using zeocin (100 μg/ml) and puromycin (2 μg/ml).

### ESC lentiviral transduction

For *ITGB1* constitutive expression, WT E14 and *Otx2* KO ESCs were transduced with the lentiviral vector, EFIa-iTGB1 (Addgene #115799) (*53*), or pLV-EF1a-IRES-Hygro (Addgene #85134) (*54*) as a negative control. To produce lentiviral particles, human embryonic kidney 293T cells were plated in a 10-cm dish. They were transfected with 3-μg lentiviral vector, along with 2-μg packaging plasmids pMDG2 and pCRV Gag-Pol (courtesy of L. James, MRC Laboratory of Molecular Biology), using 30 μl of polyethylenimine (made in-house) dissolved in Fc base medium. The next day, the medium was changed to fresh Fc base.

Seventy-two hours after transfection, the viral supernatant was filtered at 0.45 μm and collected for ESC infection. ESCs were plated in one well of a 12-well plate, 1 day before infection. They were then cultured overnight with the viral supernatant in Fc base medium, supplemented with 2i/LIF. The next day, the medium was changed to fresh Fc2iL. Antibiotic selection was then carried out on infected ESCs for 7 days, using hygromycin (200 μg/ml).

EFIa-iTGB1 was deposited to Addgene as a gift from J. Massague (Memorial Sloan Kettering Cancer Center). pLV-EF1a-IRES-Hygro was deposited to Addgene as a gift from T. Meyer (Stanford University).

### EB treatments

The “Matrigel addition time course” was carried out by adding growth factor–reduced Matrigel (354230, Corning) to different samples on different days of the 4-day time course. Before adding Matrigel to a given sample of EBs, 50 μl of PGC medium was carefully aspirated from each well of the 96-well plate. A total of 10% Matrigel was dissolved in 50 μl of cold PGC medium and added to the remaining 50 μl of PGC medium in each well. The EBs were then cultured at 37°C for the remainder of the 4-day culture.

The “Matrigel removal time course” was carried out by adding growth factor–reduced Matrigel (354230, Corning) to all samples at day 0 and subsequently removing it on different days of the 4-day time course. To remove Matrigel from a given sample of EBs, a partial ECM digestion was carried out by washing the EBs with PBS, adding dispase (07923, STEMCELL Technologies) in DMEM/F-12, and incubating for 3 min at 37°C. The EBs were subsequently washed again with PBS, resuspended in fresh PGC medium, and plated in a 12-well suspension culture plate for the remainder of the 4-day culture.

Various ECM components were added to preaggregated EBs, which were preseeded in 50 μl of PGC medium per well, following the same protocol for PGCLC induction outlined above. These components were added at twofold concentration in 50 μl of cold PGC medium, bringing the total volume per well to 100 μl and diluting them to their required final concentrations. The twofold concentrations used in 50 μl of cold PGC medium were 10% growth factor–reduced Geltrex (Gibco, A1413302), laminin-entactin (0.54 mg/ml; Corning, 354259), iMatrix-111 recombinant laminin E8 fragment (laminin-111) (0.54 mg/ml; AMSBIO, AMS.892 071), iMatrix-511 recombinant laminin E8 fragment (laminin-511) (0.54 mg/ml; Takara Bio, T303), collagen IV (0.27 mg/ml; Corning, 354233), collagen I (0.018 mg/ml; Sigma-Aldrich, C3867), and fibronectin (0.018 mg/ml; 1918-FN, R&D Systems).

To induce E-cadherin–GFP overexpression in Dox-inducible E-cadherin–GFP EBs, Dox hyclate (D9891, Sigma-Aldrich) was added to PGC medium at 1 or 10 μg/ml. To activate Wnt signaling in Prdm14::mVenus EBs, a GSK3 inhibitor (CHIR99021, Cambridge Stem Cell Institute) was added to PGC medium at 3 μM. Alternatively, recombinant Wnt3a (1324-WN, R&D Systems) was added to PGC medium at varying concentrations. To inhibit Ilk signaling in Prdm14::mVenus EBs, an Ilk inhibitor (Cpd 22, 407331, Merck) was added to PGC medium at 100 nM. To inhibit Src signaling in Prdm14::mVenus EBs, a Src inhibitor (Dasatinib, Cayman Chemical, 11498) was added to PGC medium at 1 μM. All treatments were carried out for the entire 4-day duration of PGCLC induction.

### Immunofluorescence

Both embryos and cells were fixed using 4% paraformaldehyde (15710, Electron Microscopy Sciences) diluted in PBS. Samples were fixed for 20 to 30 min at room temperature and then washed three times with PBS–0.1% Tween. For permeabilization, embryos were incubated in a permeabilization buffer (PBS, 0.5% Triton X-100, and 0.1 M glycine) for 30 min to 1 hour at room temperature. Cells were incubated in a permeabilization buffer (PBS, 0.3% Triton X-100, and 0.1 M glycine) for 20 min at room temperature. For blocking, embryos were incubated in a blocking buffer (PBS, 3% BSA, and 0.1% Tween) for at least 4 hours at room temperature or overnight at 4°C. Cells were incubated in the blocking buffer for only 30 min at room temperature. The samples were then incubated with primary antibodies (table S1), diluted in the blocking buffer, overnight at 4°C. The following day, samples were washed three times with PBS–0.1% Tween and were then incubated with secondary antibodies (table S2), diluted in the blocking buffer, for 2 hours at room temperature or overnight at 4°C. 4′,6-Diamidino-2-phenylindole (DAPI; D1306, Thermo Fisher Scientific) was used as a nuclear DNA counterstain (1:1000 dilution). All samples were cleared before imaging by incubating in 0.02 M phosphate buffer for 5 min at room temperature, air-drying for 5 to 10 min. Refractive Index Matching Solution (RIMS) buffer (*55*), composed of Histodenz (2 g/ml; D2158, Sigma-Aldrich) dissolved in 0.02 M phosphate buffer (pH 7.4), was then directly added. Alternatively, to prevent collapse of the proamniotic cavity in embryo samples, RIMS buffer was progressively added at increasing concentrations of 0.04, 0.2, and 1 g/ml Histodenz (D2158, Sigma-Aldrich) dissolved in 0.02 M phosphate buffer (pH 7.4) while incubating the sample on a shaker for 5 to 10 min at each concentration. Once the final RIMS buffer was added, all samples were then incubated at 4°C for at least 1 hour before imaging. Images were acquired on a TCS SP8 3X gated stimulated emission depletion microscopy (STED) confocal inverted microscope (Leica Microsystems) with a “Leica 40×/1.1 numerical aperture water” objective. Image acquisition and laser power settings were kept constant to allow comparison across samples within the same experiment.

### Flow cytometry

Prdm14::mVenus or Blimp1::mGFP EBs were dissociated into single cells by washing them with PBS, before adding TrypLE and incubating them for 5 min at 37°C. After incubation, agitation by pipetting allowed us to obtain a single-cell suspension, which was centrifuged for 4 min at 300*g*, washed with PBS, and centrifuged again. The pellet of single cells was resuspended in a fluorescence-activated cell sorting (FACS) buffer (PBS–2% FBS).

Flow cytometry analysis was performed using a CytoFlex LX Flow Cytometer (Beckman Coulter). Propidium iodide (1304MP, Thermo Fisher Scientific) was added (50 μg/ml) to the single-cell suspension as a viability dye and used to gate live cells. GFP was measured for all live cells, and WT E14 cells were used as a negative control to gate GFP^+^ cells. The gating strategy is illustrated in fig. S2A. PGCLC specification efficiency was defined as the percentage of GFP^+^ cells in a given sample. All values were normalized by the mean percentage of GFP^+^ cells in the M − samples for the same experiment.

FACS of GFP^+^ cells from Blimp1::mGFP EBs was performed using a Bigfoot Spectral Cell Sorter (Thermo Fisher Scientific) with a nozzle size of 100 μm and a pressure of approximately 200 KPa. GFP was measured for all cells, and WT E14 cells were used as a negative control to gate GFP^+^ cells. Both GFP^+^ and GFP^−^ cells were collected for subsequent RNA extraction and gene expression analysis by quantitative reverse transcription polymerase chain reaction (qRT-PCR).

### RNA extraction and qRT-PCR

For day 2 and day 4 EBs, as well as for GFP^−^ and GFP^+^ populations sorted from day 2 and day 4 Blimp1::mGFP EBs, RNA was extracted using the PicoPure RNA Isolation Kit (KKIT0204, Thermo Fisher Scientific), following the manufacturer’s instructions. Subsequently, 500 ng of RNA was used to perform a reverse transcription reaction. The reaction comprised random primers (C1181, Promega), deoxyribonucleotide triphosphates (N0447S, New England Biolabs), M-MuLV reverse transcriptase (M0253L, New England Biolabs), and ribonuclease inhibitor (M0314L, New England Biolabs). qRT-PCR reactions were performed using Power SYBR Green PCR Master Mix (4309155, Thermo Fisher Scientific) and run on a ViiA 7 Real-Time PCR machine (Thermo Fisher Scientific). The run method consisted of an initialization step (95°C, 10 min), followed by 40 cycles of both a denaturation step (95°C, 15 s) and an annealing and extension step (60°C, 1 min). The primers used are listed in table S3. Gene expression data were normalized to Gapdh. All gene expression levels were reported relative to control (M −) levels at the same time point and in the same experiment, unless otherwise stated.

### Live-cell imaging

Live-cell imaging was done using the Incucyte Live-Cell Analysis System (Sartorius). Both “Phase + Brightfield” and “Green” image channels were activated, and image acquisition was done using the “Spheroid” scan type and S3/SX1 GR optical module, at ×4 magnification and 300-ms acquisition time. Images were acquired at 6-hour intervals for 4 days. Images were then exported and analyzed in Fiji.

### Image analysis

Embryos were visualized in 3D using Imaris software. The normalized PGC coverage in embryos was determined on the basis of the percentage area coverage of Blimp1::mGFP^+^ Nanog^+^ or Blimp1::mGFP^+^ Ap2γ^+^ cells. This was calculated by manually labeling regions of interest (ROIs) from 3D z-stacks in Fiji. To correct for differences in size across embryos, we identified a single plane with the largest area of Nanog^+^ epiblast cells and used this as a proxy for size in each embryo. The total Blimp1::mGFP^+^ Nanog^+^ or Blimp1::mGFP^+^ Ap2γ^+^ area was divided by the area of the epiblast for a given embryo. All values were normalized by the mean percentage area coverage of all control samples in the same experiment. In all experiments, embryos with abnormal morphologies were excluded from the analysis.

Normalized β1 integrin, P-Fak-Tyr(576), and Otx2 levels in embryos were calculated by identifying Blimp1::mGFP^+^ Nanog^+^ cells, specifically in planes where at least one of the cells was in contact with the basement membrane. To calculate β1 integrin and P-Fak-Tyr(576) levels for any given cell, a “smoothing” function was applied to the image, and a line segment was drawn perpendicular across the side of the cell expressing the highest intensity of β1 integrin or P-Fak-Tyr(576). A fluorescence intensity plot was generated along the line segment, using the “Plot Profile” function in Fiji, and the area under the peak of the curve was taken as a measure of β1 integrin or P-Fak-Tyr(576) levels for that cell. Otx2 levels were calculated for a given cell by manually labeling nuclear ROIs and calculating the mean Otx2 fluorescence intensity for each nuclear ROI. Normalized β1 integrin levels were then determined by normalizing β1 integrin and levels for each cell by the mean β1 integrin level of all cells in the same plane that were not in contact with the basement membrane. Normalized P-Fak-Tyr(576) and Otx2 levels were determined by normalizing P-Fak-Tyr(576) or Otx2 levels for each cell by the mean P-Fak-Tyr(576) or Otx2 levels, respectively, of all cells in the same plane that were both in contact with the basement membrane and did not express Blimp1::mGFP. In addition, a line profile was generated using the Plot Profile function in Fiji. The line was drawn perpendicular to the membrane in a Blimp1::mGFP^+^ cell in contact with the basement membrane, a Blimp1::mGFP^−^ cell in contact with the basement membrane, and a Blimp1::mGFP^+^ cell not in contact with the basement membrane.

The aspect ratio of MMP inhibitor–treated embryos was calculated by first identifying the plane in the 3D z-stack with the longest longitudinal dimension. The aspect ratio was then calculated as the length of the longitudinal dimension divided by the width of the epiblast, both measured as line segments in Fiji.

Expression values in EBs were calculated from 3D z-stacks, by measuring the area of a binary mask for the relevant marker across all planes and dividing it by the area of the nuclear (DAPI) mask. PGCLC specification efficiency in EBs was quantified by calculating the expression value for the relevant PGCLC marker(s) (Blimp1::mGFP, Oct4-Nanog-Ap2γ, or Nanog-Ap2γ). In the case of multiple markers (Oct4-Nanog-Ap2γ and Nanog-Ap2γ), the expression value was calculated by finding the overlap between the masks of the individual markers. The nuclear (DAPI) mask was used to quantify the size of embryos and EBs, by multiplying the size of the mask by the voxel depth, width, and height. All EB values were normalized by the mean value of all control M − samples in the same experiment, unless otherwise stated. For time course immunostaining experiments, EB values were normalized by the mean value of all control M − samples on day 4 in the same experiment.

To analyze the correlation, within EBs, between levels of Brachyury and Otx2 in individual cells, a representative plane in the 3D z-stack of an EB was first selected. Next, the DAPI image was smoothed using the “Gaussian Blur” function in Fiji, setting the variance to 2. The binary threshold was then manually adjusted to capture binary nuclear segments at approximately single-nucleus resolution, before applying a “watershed” function to the binary mask. The “Analyze Particles” function was used to generate an ROI set of segmented nuclei, which was separately overlaid on both the Brachyury and Otx2 images to then measure the mean intensity of the two markers in each nucleus. The mean intensity values of each marker were normalized to the maximum mean intensity value among all cells in each plane, to represent all intensities as values between 0 and 1. These normalized values were then plotted on an *XY* plot. Only single- and double-positive nuclei, expressing either Brachyury and/or Otx2, were considered for the analysis, by manually setting a threshold based on the distribution of normalized intensity values. The number of PGCs per embryo was manually counted using the “Cell Counter” Fiji plugin.

Mean fluorescence intensity values in live-cell images were quantified, for each sample and time point, by segmenting EBs in the brightfield image and overlaying the brightfield segmentation onto the corresponding fluorescent image. The mean fluorescence intensity was calculated within the area bounded by the brightfield segmentation.

### Statistical analyses

All statistical analyses were performed using GraphPad Prism. Embryos were randomly allocated to control and experimental groups while trying to maintain an equal representation of different sizes, stages, and morphologies across both groups. The sample size was determined on the basis of previous experimental experience, and investigators were not blind to group allocation. Qualitative data are shown as a contingency bar graph and were analyzed using a Fisher’s exact test (two groups). Quantitative data are shown as mean ± SEM. Box-and-whisker plots are shown as mean with minimum and maximum. Violin plots are shown as mean and interquartile range (IQR). The normality of the data was analyzed using a Kolmogorov-Smirnov test. Data that did not follow a Gaussian distribution were analyzed with a Mann-Whitney *U* test (two groups) or a Kruskal-Wallis test (multiple groups). Data that followed a Gaussian distribution were analyzed with an unpaired Student’s *t* test (two groups) or an ordinary one-way analysis of variance (ANOVA) test (multiple groups). An *F* test was used to determine whether the variances between groups were significantly different, and a Welch’s correction was applied accordingly. All statistical tests were two-sided unless otherwise stated.

*Note added in proof*: After the manuscript was accepted for publication, the authors requested that the readers be made aware of an additional paper to reflect the current literature (*56*).
