# Supplementary material for: Integrin signaling in pluripotent cells acts as a gatekeeper of mouse germline entry
Source: Sci Adv. 2024 Sep 4;10(36):eadk2252. doi: 10.1126/sciadv.adk2252 (PMC11373592; doi:10.1126/sciadv.adk2252)
Supplement: Supplementary file 1 — Figs. S1 to S5 Tables S1 to S3 Legend for movie S1 [file sciadv.adk2252_sm.pdf]

Science Advances
AAAS

Supplementary Materials for

Integrin signaling in pluripotent cells acts as a gatekeeper of mouse germline entry

Aly Makhlouf *et al.*

Corresponding author: Marta N. Shahbazi, mshahbazi@mrc-lmb.cam.ac.uk

Sci. Adv. **10**, eadk2252 (2024)
DOI: 10.1126/sciadv.adk2252

The PDF file includes:

Figs. S1 to S5
Tables S1 to S3
Legend for movie S1

Other Supplementary Material for this manuscript includes the following:

Movie S1

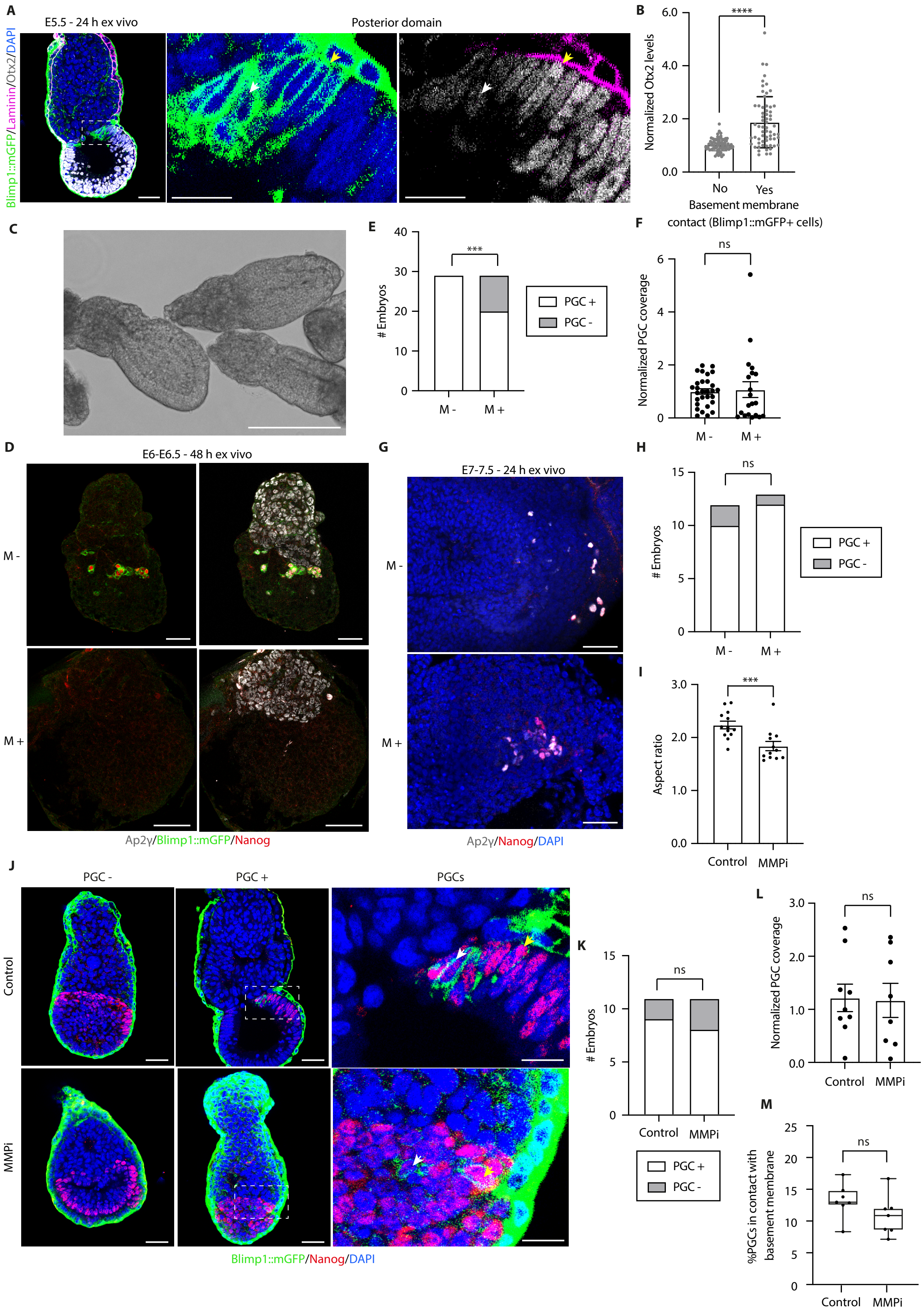
A
E5.5 - 24 h ex vivo
Posterior domain
Blimp1::mGFP/Laminin/Otx2/DAPI
B
****
Normalized Otx2 levels
No
Yes
Basement membrane contact (Blimp1::mGFP+ cells)
C
E
***
Embryos
PGC +
PGC -
M -
M +
F
ns
Normalized PGC coverage
D
E6-E6.5 - 48 h ex vivo
Ap2γ/Blimp1::mGFP/Nanog
G
E7-7.5 - 24 h ex vivo
Ap2γ/Nanog/DAPI
H
ns
I
***
Aspect ratio
Control
MMPi
J
PGC -
PGC +
PGCs
Blimp1::mGFP/Nanog/DAPI
K
L
M
%PGCs in contact with basement membrane

Figure S1: PGC specification in *ex vivo* cultured embryos. (A) Immunostaining of embryos cultured *ex vivo* for 24 hours. **(B)** Otx2 levels of Blimp1::mGFP+ cells classified based on basement membrane contact in embryos from A. Data are shown as mean ± SEM. Each point represents an individual cell. n = 75 (No) and n = 63 (Yes) cells. 5 embryos. 2 independent experiments. Mann Whitney U test. ****$p < 0.0001$. **(C)** Brightfield image of E6.25 Blimp1::mGFP reporter mouse embryos devoid of VE. Scale bars: 200 µm. **(D)** Immunostaining of embryos devoid of VE and cultured *ex vivo* for 48 hours, without (M -) or with (M +) 5% dissolved Matrigel. **(E)** Number of embryos from D that have (PGC +) or have not (PGC -) specified Blimp1::mGFP+ Nanog+ PGCs. Data are shown as a contingency bar graph. n = 29 (M -) and n = 29 (M +) embryos. 10 independent experiments. Fisher's exact test. ***$p = 0.0009$. **(F)** Area coverage of Blimp1::mGFP+ cells in embryos from panel D. Only embryos that had PGCs were selected for this analysis. Data are shown as mean ± SEM. Each point represents an embryo. n = 29 (M -) and n = 20 (M +) embryos. 10 independent experiments. Welch's t-test. ns: non-significant. **(G)** Immunostaining of E7.5 embryos devoid of VE and cultured *ex vivo* for 24 hours, without (M -) or with (M +) 5% dissolved Matrigel. **(H)** Number of embryos from G that have (PGC +) or have not (PGC -) specified Blimp1::mGFP+ Nanog+ PGCs. Data are shown as a contingency bar graph. n = 12 (M -) and n = 13 (M +) embryos. 2 independent experiments. Fisher's exact test. ns: non-significant. **(I)** Aspect ratio of embryos from J. Data are shown as mean ± SEM. Each point represents an embryo. n = 13 (Control) and n = 12 (MMPi) embryos. 4 independent experiments. Mann-Whitney test. ***$p = 0.001$. **(J)** Immunostaining of E5.5 mouse embryos cultured *ex vivo* for 24 hours, with or without MMP inhibitors (MMPi). **(K)** Number of embryos from J that have (PGC +) or have not (PGC -) specified Blimp1::mGFP+Nanog+ PGCs. Data are shown as a contingency bar graph. n = 11 (Control) and n = 11 (MMPi) embryos. 4 independent experiments. Fisher's exact test. ns: non-significant. **(L)** Area coverage of Blimp1::mGFP+ cells in embryos from J. Data are shown as mean ± SEM. Each point represents an embryo. n = 9 (Control) and n = 8 (MMPi) embryos. 4 independent experiments. Welch's t-test. ns: non-significant. **(M)** Percentage of Blimp1::mGFP+ cells in contact with the basement membrane in embryos from J. Data are shown as a box-and-whisker plot (mean with minimum and maximum). Each point represents an embryo. n = 7 (Control) and n = 7 (MMPi) embryos. 3 independent experiments. Unpaired Student's t-test. ns: non-significant. For all panels, scale bars: 50 µm, 20 µm (magnified regions, which are indicated with a dashed white box). Yellow arrows indicate Blimp1::mGFP+ cells in contact with the basement membrane, and white arrows Blimp1::mGFP+ cells not in contact. MMPi: Matrix metalloproteinase inhibitors.

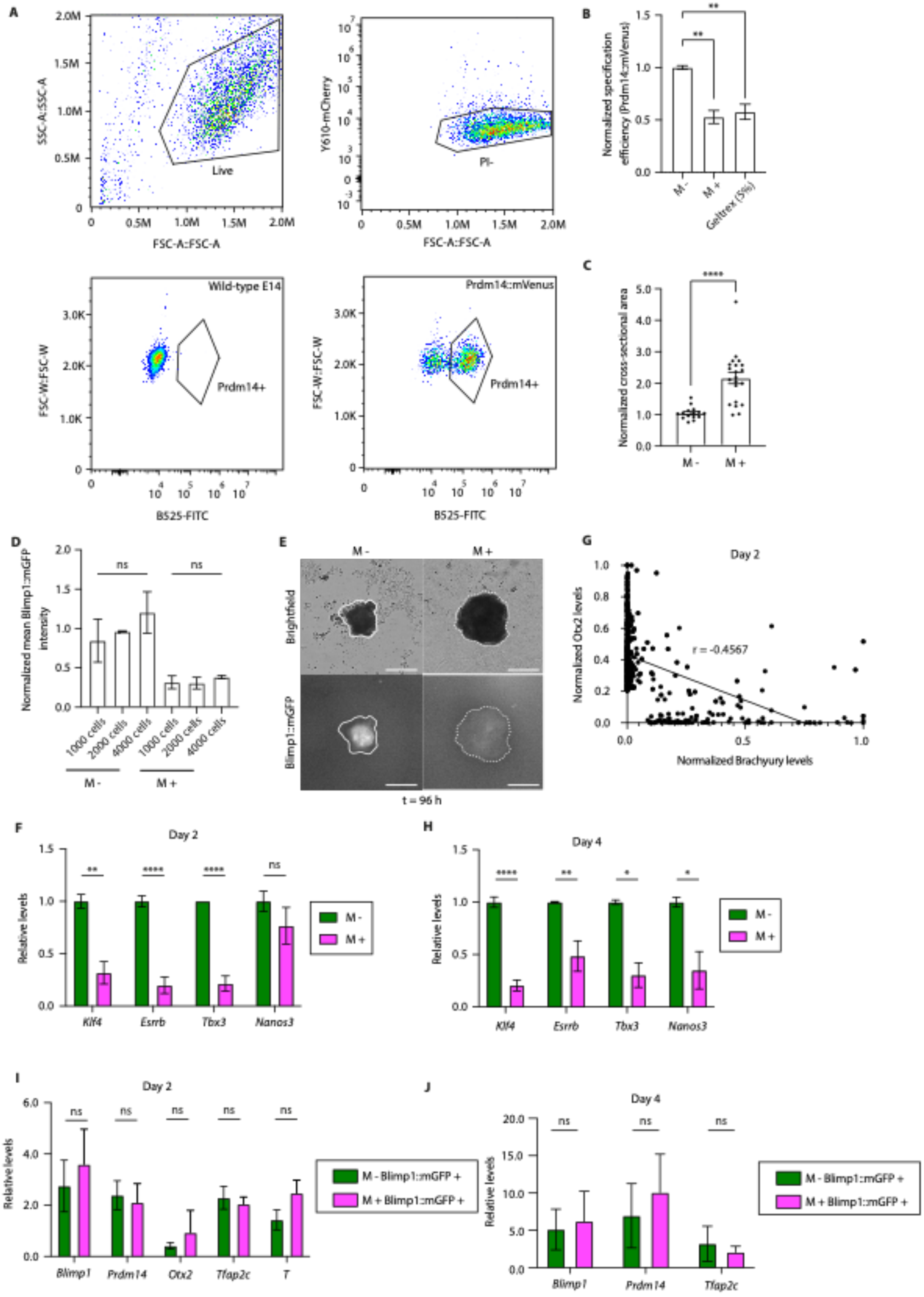

A
Live
PI-
Wild-type E14
Prdm14::mVenus
Prdm14+
B525-FITC
FSC-A::FSC-A
SSC-A::SSC-A
Y610-mCherry
FSC-W::FSC-W
B
Normalized specification efficiency (Prdm14::mVenus)
M -
M +
Geltrex (5%)
C
Normalized cross-sectional area
D
Normalized mean Blimp1::mGFP intensity
1000 cells
2000 cells
4000 cells
ns
E
Brightfield
Blimp1::mGFP
t = 96 h
G
Day 2
Normalized Otx2 levels
Normalized Brachyury levels
r = -0.4567
F
Day 2
Relative levels
Klf4
Esrrb
Tbx3
Nanos3
H
Day 4
I
Day 2
Blimp1
Prdm14
Otx2
Tfap2c
T
M - Blimp1::mGFP +
M + Blimp1::mGFP +
J
Day 4

Figure S2: ECM inhibits PGCLC specification. (A) Gating strategy for flow cytometry analysis. The top left panel shows the initial gating for live cells, using forward and side scatters. The top right panel shows an additional gating, selecting for live cells that are unstained for propidium iodide. The bottom panels show the final gating for single cells dissociated from Prdm14::mVenus+ EBs (bottom right), using wild-type E14 EBs that do not express the mVenus reporter as a negative control (bottom left). **(B)** PGCLC specification efficiency (analyzed by flow cytometry) in Prdm14::mVenus reporter EBs, cultured without (M -) or with 5% dissolved Matrigel (M +), or with 5% dissolved Geltrex. Data are shown as mean ± SEM. n = 7 samples. 3 independent experiments. One-way ANOVA test with Welch's correction. **p = 0.0019 (M +), **p = 0.0090 (Geltrex 5%). **(C)** Cross-sectional area of EBs, cultured without (M -) or with (M +) 5% dissolved Matrigel. Data are shown as mean ± SEM. Each point represents an EB. n = 16 (M -) and n = 23 (M +) EBs. 6 independent experiments. Welch's t-test. ****p < 0.0001. **(D)** Mean Blimp1::mGFP intensity (analyzed by live-cell imaging) in reporter EBs aggregated from variable numbers of seeded cells. Data are shown as mean ± SEM. n = 2 samples. 2 independent experiments. Kruskal-Wallis test. ns: non-significant. **(E)** Brightfield and fluorescence (Blimp1::mGFP) images acquired for live-cell imaging analysis using Incucyte®. Dashed lines indicate the brightfield segmentation used to quantify mean fluorescence intensities in EBs. Scale bars, 100 µm. **(F)** Time-course gene expression analysis (qRT-PCR) of EBs on day 2, cultured without (M -) or with (M +) 5% dissolved Matrigel. Data are shown as mean ± SEM. n = 6 samples. 3 independent experiments. Unpaired Student's t-test. 0.01 < *p < 0.05, 0.001 < **p < 0.01, 0.0001 < ***p < 0.001, ****p < 0.0001, and ns: non-significant. **(G)** XY plot of Brachyury levels vs. Otx2 levels in single cells from EBs on day 2. n = 721 cells. 4 independent experiments. Simple linear regression. **(H)** Time-course gene expression analysis (qRT-PCR) of EBs on day 4, cultured without (M -) or with (M +) 5% dissolved Matrigel. Data are shown as mean ± SEM. n = 6 samples. 3 independent experiments. Unpaired Student's t-test. 0.01 < *p < 0.05, 0.001 < **p < 0.01, 0.0001 < ***p < 0.001, ****p < 0.0001, and ns: non-significant. **(I-J)** Time-course gene expression analysis (qRT-PCR) of Blimp1::mGFP+ cells sorted from EBs on day 2 **(I)** and day 4 **(J)**, cultured without (M -) or with (M +) 5% dissolved Matrigel. All values are reported relative to corresponding gene expression levels in Blimp1::mGFP- cells from the same time-point (day 2 or day 4), under the same condition (M - or M +), and in the same experiment. Data are shown as mean ± SEM. n = 5 samples. 5 independent experiments. Mann-Whitney U test. ns: non-significant.

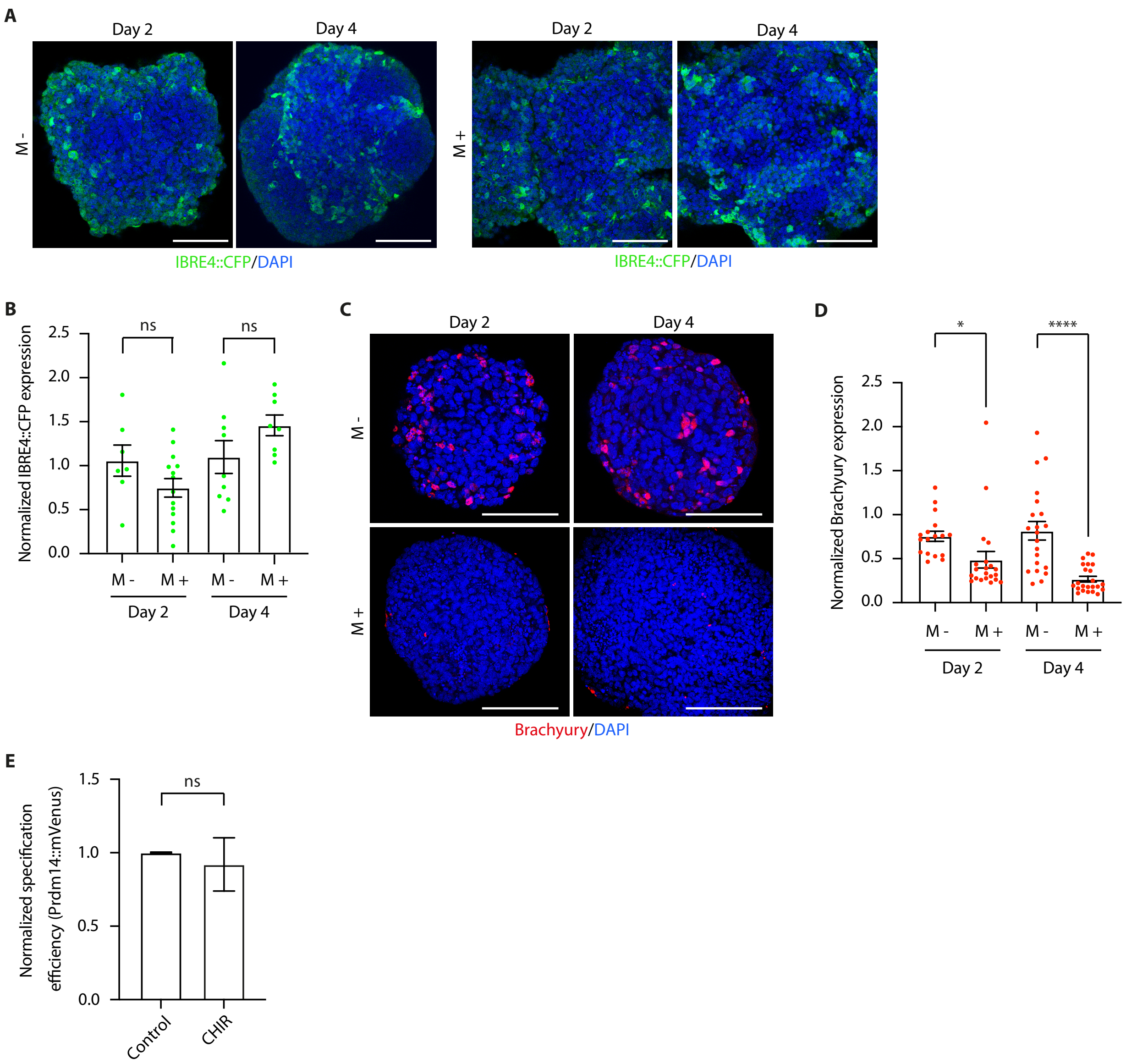

Figure S3: ECM inhibits Wnt but not Bmp signaling. (A) Time-course immunostaining of Bmp reporter EBs (IBRE4::CFP), cultured without (M -) or with (M +) 5% Matrigel. **(B)** Bmp activity in reporter EBs from A. Data are shown as mean ± SEM. Each point represents an individual EB. n = 7 (M - Day 2), n = 14 (M + Day 2), n = 9 (M - Day 4), and n = 8 (M + Day 4) EBs. 2 independent experiments. Ordinary one-way ANOVA test. ns: non-significant. **(C)** Time-course immunostaining of Brachyury in EBs, cultured without (M -) or with (M +) 5% Matrigel. **(D)** Brachyury expression in EBs from panel C. Data are shown as mean ± SEM. Each point represents an EB. n = 17 (M - Day 2), n = 21 (M + Day 2), n = 21 (M - Day 4), and n = 23 (M + Day 4) EBs. 5 independent experiments. Kruskal-Wallis test. *p = 0.0102, and ****p < 0.0001. **(E)** PGCLC specification efficiency (analyzed by flow cytometry) in Prdm14::mVenus reporter EBs, cultured with or without a Wnt activator (3 µM CHIR99021). Data are shown as mean ± SEM. n = 4

samples. 2 independent experiments. Mann-Whitney U test. ns: non-significant. For all panels, scale bars: 100 μm.

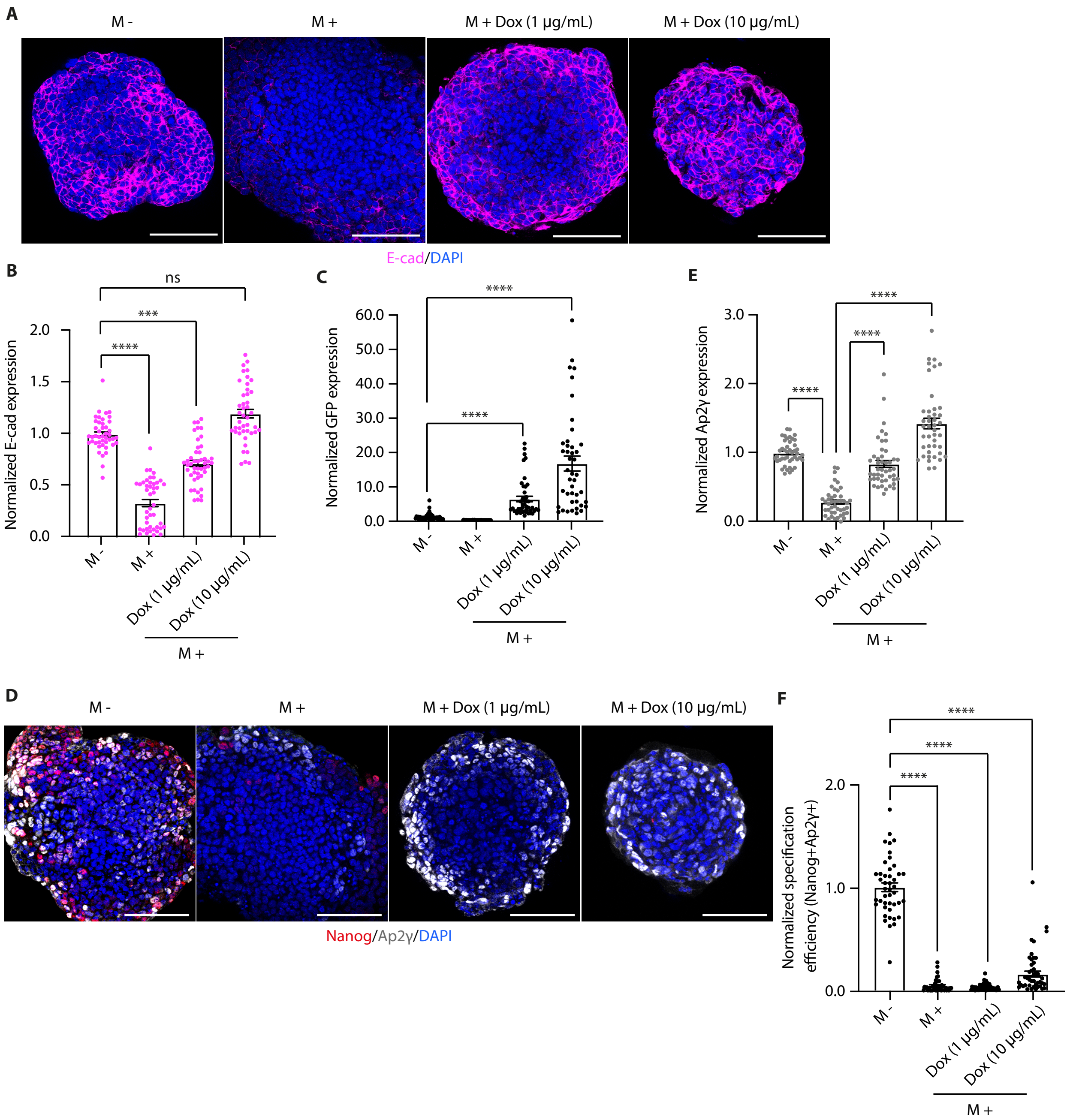

Figure S4: E-cadherin overexpression does not restore PGCLC specification inhibited by ECM. (A) Immunostaining of doxycycline (Dox)-inducible E-cadherin-GFP-overexpressing EBs, cultured without (M -) or with (M +) 5% Matrigel. **(B)** E-cadherin expression in EBs from A. Data are shown as mean ±SEM. Each point represents an EB. n = 44 (M -), n = 44 (M +), n = 46 (M + Dox 1 µg/ml), and n = 43 (M + Dox 10 µg/ml) EBs. 4 independent experiments. Kruskal-Wallis test. ***$p = 0.0003$, ****$p < 0.0001$, and ns: non-significant. **(C)** GFP expression in EBs from pA. Data are shown as mean ± SEM. Each point represents an EB. n = 44 (M -), n = 44 (M +), n = 46 (M + Dox 1 µg/mL), and n = 43 (M + Dox 10 µg/mL) EBs. 4 independent experiments. Kruskal-Wallis test. ****$p < 0.0001$. **(D)** Immunostaining of doxycycline (Dox)-inducible E-cadherin-

GFP-overexpressing EBs, cultured without (M -) or with (M +) 5% Matrigel. **(E)** AP2γ expression in EBs from D. Data are shown as mean ± SEM. Each point represents an EB. n = 44 (M -), n = 44 (M +), n = 46 (M + Dox 1 μg/mL), and n = 43 (M + Dox 10 μg/mL) EBs. 4 independent experiments. Kruskal-Wallis test. ****$p < 0.0001$. **(F)** PGCLC specification efficiency in EBs from D. Data are shown as mean ± SEM. Each point represents an EB. n = 44 (M -), n = 44 (M +), n = 46 (M + Dox 1 μg/mL), and n = 43 (M + Dox 10 μg/mL) EBs. 4 independent experiments. Kruskal-Wallis test. ****$p < 0.0001$. For all panels, scale bars: 100 μm.

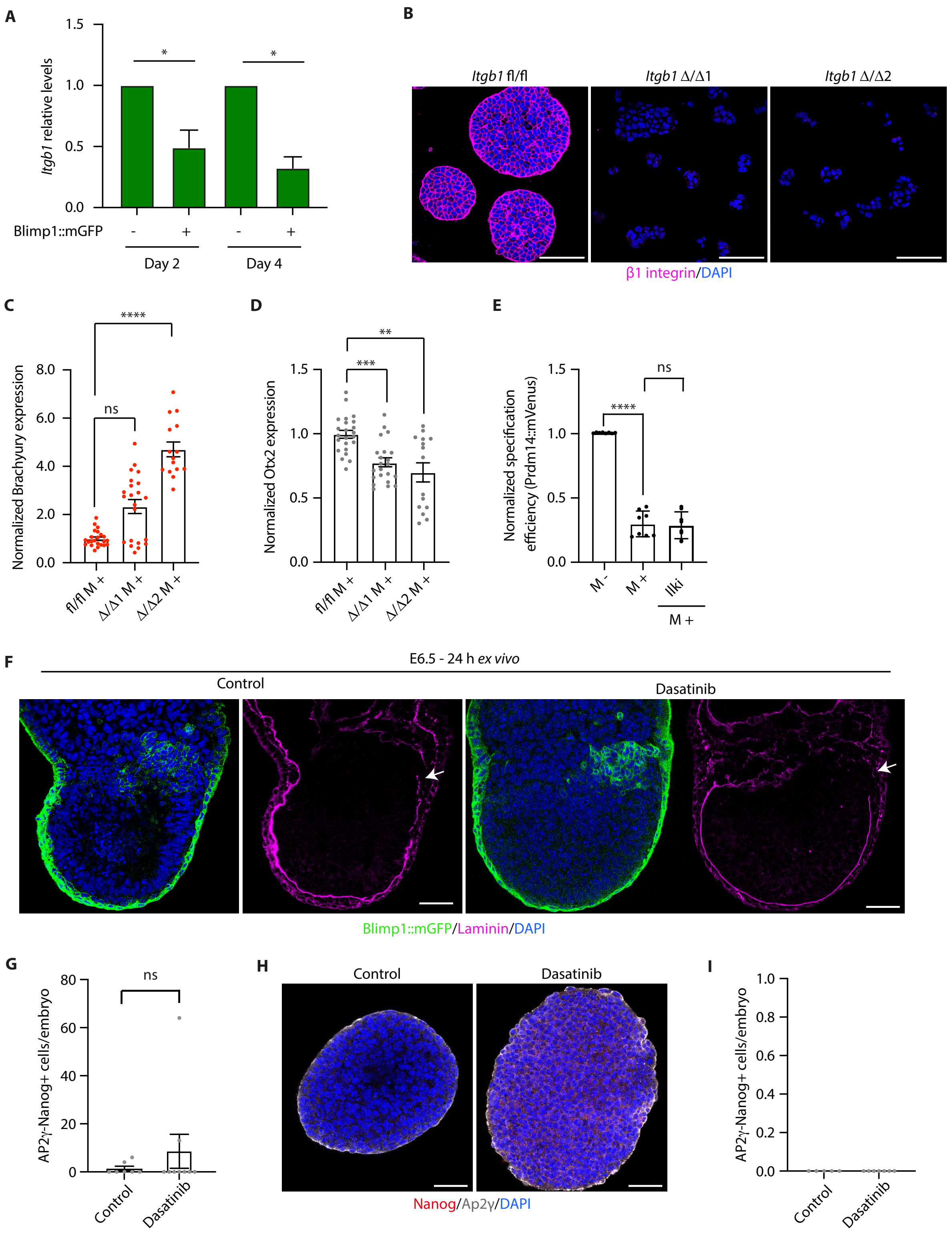

Figure S5: β1 integrin inhibits germline entry. (A) Time-course gene expression analysis (qRT-PCR) of *Itgb1* levels in Blimp1::mGFP- and Blimp1::mGFP+ cells sorted from EBs on day 2 and day 4. All values are reported relative to corresponding gene expression levels in Blimp1::mGFP-

cells from the same time-point (day 2 or day 4) and in the same experiment. Data are shown as mean ± SEM. n = 5 samples. 5 independent experiments. Kruskal-Wallis test. *p = 0.0475 (Day 2), *p = 0.0135 (Day 4). **(B)** Immunostaining of control (fl/fl) and *Itgb1* KO (Δ/Δ) ESCs. Scale bars: 100 µm. **(C)** Brachyury levels in EBs from Figure 4H. Data are shown as mean ± SEM. Each point represents an EB. n = 23 (fl/fl M +), n = 22 (Δ/Δ1 M +), and n = 15 (Δ/Δ2 M +) EBs. 3 independent experiments. Kruskal-Wallis test. ****p < 0.0001 and ns: non-significant. **(D)** Otx2 expression in EBs from Figure 4H. Data are shown as mean ± SEM. Each point represents an EB. n = 23 (fl/fl M +), n = 22 (Δ/Δ1 M +), and n = 15 (Δ/Δ2 M +) EBs. 3 independent experiments. Kruskal-Wallis test. **p = 0.0019, and ***p = 0.0009. **(E)** PGCLC specification efficiency (analyzed by flow cytometry) in Prdm14::mVenus reporter EBs, cultured without (M -) or with (M +) 5% dissolved Matrigel and adding 100 nM of the ILK inhibitor (ILKi) Cpd22. Data are shown as mean ± SEM. Each point represents an independent sample. n = 8 samples. 4 independent experiments. Ordinary one-way ANOVA test. ****p < 0.0001 and ns: non-significant. **(F)** Immunostaining of E6.5 embryos, cultured *ex vivo* for 24 hours. Scale bars: 50 µm. **(G)** Number of PGCs (Nanog/AP2γ+ cells) in epiblast-VE fragments from Figure 6I. Data are shown as mean ± SEM. Each point represents an epiblast-VE fragment. n = 7 (Control) and n= 9 (Dasatinib) epiblast-VE fragments. Mann-Whitney test. ns: non-significant. **(H)** Isolated epiblasts from E6.5 embryos cultured *in vitro* for 48 hours without (Control) or with a Src inhibitor (Dasatinib). **(I)** Number of PGCs (Nanog/AP2γ+ cells) in epiblasts from H. Each point represents an epiblast. n = 5 (Control) and n = 7 (Dasatinib) epiblasts.

Table S1: Primary antibodies used for immunostaining

Antibody	Type	Cat. #	Company	Dilution	Use
Mouse Anti-Ap2γ	Monoclonal, 6E4/4	sc-53162	Santa Cruz Biotechnology	1:200	Whole-mount embryo staining
Goat Anti-Ap2γ	Polyclonal	AF5059	Novus Biologicals	1:200	EB staining
Goat Anti-Brachyury	Polyclonal	AF2085	R&D Systems	1:300	EB staining
Rabbit Anti-Brachyury	Monoclonal, D2Z3J	81694	Cell Signaling Technology	1:1000	EB staining
Rat Anti-E-Cadherin	Monoclonal, ECCD-2	13-1900	Thermo Fisher Scientific	1:200	EB staining
Chicken Anti-GFP	Polyclonal	ab13970	Abcam	1:1000	EB and whole-mount embryo staining
Rat Anti-β1 Integrin	Monoclonal, MB1.2	MAB1997	Merck	1:200	EB and whole-mount embryo staining
Rat Anti-β1 Integrin (Human)	Monoclonal, mAb13	MABT821	Merck	1:200	EB staining
Rabbit Anti-Laminin	Polyclonal	L9393	Sigma-Aldrich	1:400	EB staining
Rabbit Anti-Nanog	Polyclonal	ab80892	Abcam	1:200	EB and whole-mount embryo staining

Antibody	Type	Cat. #	Company	Dilution	Use
Mouse Anti-Oct4	Monoclonal, C-10	sc-5279	Santa Cruz Biotechnology	1:100	EB staining
Goat Anti-Otx2	Polyclonal	AF1979	R&D Systems	1:200	EB and whole-mount embryo staining
Rabbit anti-phospho-Fak-Tyr(576)	Monoclonal, 2H74L24	700013	Invitrogen	1:100	Whole-mount embryo staining
Rat Anti-Podocalyxin	Monoclonal, 192703	MAB1556	R&D Systems	1:500	Whole-mount embryo staining
Goat Anti-Sox17	Polyclonal	AF1924	R&D Systems	1:200	EB staining

Table S2: Secondary antibodies used for immunostaining

Antibody	Cat. #	Company	Dilution
Donkey anti-Mouse IgG (H+L) Alexa Fluor™ 488	A21202	Thermo Fisher Scientific	1:500
Donkey anti-Rabbit IgG (H+L) Alexa Fluor™ 488	A21206	Thermo Fisher Scientific	1:500
Donkey anti-Rat IgG (H+L) Alexa Fluor™ 488	A21208	Thermo Fisher Scientific	1:500
Donkey anti-Goat IgG (H+L) Alexa Fluor™ 488	A11055	Thermo Fisher Scientific	1:500

Antibody	Cat. #	Company	Dilution
Donkey anti-Chicken IgG (H+L) Alexa Fluor™ 488	A78948	Thermo Fisher Scientific	1:500
Donkey anti-Mouse IgG (H+L) Alexa Fluor™ 555	A31570	Thermo Fisher Scientific	1:500
Donkey anti-Rabbit IgG (H+L) Alexa Fluor™ 555	A31572	Thermo Fisher Scientific	1:500
Donkey anti-Goat IgG (H+L) Alexa Fluor™ 555	A21432	Thermo Fisher Scientific	1:500
Donkey anti-Mouse IgG (H+L) Alexa Fluor™ 594	A21203	Thermo Fisher Scientific	1:500
Donkey anti-Rabbit IgG (H+L) Alexa Fluor™ Plus 594	A32754	Thermo Fisher Scientific	1:500
Donkey anti-Rat IgG (H+L) Alexa Fluor™ 594	A21209	Thermo Fisher Scientific	1:500
Donkey anti-Goat IgG (H+L) Alexa Fluor™ 594	A11058	Thermo Fisher Scientific	1:500
Donkey anti-Mouse IgG (H+L) Alexa Fluor™ Plus 647	A32787	Thermo Fisher Scientific	1:500
Donkey anti-Rabbit IgG (H+L) Alexa Fluor™ 647	A31573	Thermo Fisher Scientific	1:500
Donkey anti-Rat IgG (H+L) Alexa Fluor™ Plus 647	A48272	Thermo Fisher Scientific	1:500
Donkey anti-Goat IgG (H+L) Alexa Fluor™ 647	A21447	Thermo Fisher Scientific	1:500

Table S3: qRT-PCR primers used for gene expression analysis

Gene	5'-FW-3'	5'-RV-3'
Gapdh	ACTGGCATGGCCTTCCGTGTTC	ATTGAGAGCAATGCCAGCCCCG
Prdm1	AGCATGACCTGACATTGACACC	CTCAACACTCTCATGTAAGAGGC
Prdm14	ACAGCCAAGCAATTTGCACTAC	TTACCTGGCATTTTCATTGCTC
Dppa3	AGGCTCGAAGGAAATGAGTTTG	TCCTAATTCTTCCCGATTTTCG
Pou5f1	TGCAAATCGGAGACCCTGGTGC	ATCTTTTGCCCTTCTGGCGCCG
Sox2	GCTCGCAGACCTACATGAAC	GCCTCGGACTTGACCACAG
Nanog	TCTTCCTGGTCCCCACAGTTT	GCAAGAATAGTTCTCGGGATGAA
Cdh1	CTCCAGTCATAGGGAGCTGTC	TCTTCTGAGACCTGGGTACAC
T	GCTTCAAGGAGCTAACTAACGAG	CCAGCAAGAAAGAGTACATGGC
Otx2	TATCTAAAGCAACCGCCTTACG	GCCCTAGTAAATGTCGTCCTCTC
Klf4	TCGTGGCCCCGGAAAAGAACAG	TGTGTGAGTTCGCAGGTGTGCC
Esrrb	TTTGTGCCATAGGAGGGCAG	GGGCTGAGGGGTTTTAGGAC
Tbx3	TTTTGTCTGGGAGGGAGCAC	ACTTCCATACCAGCCTGCAC
Nanos3	CACTACGGCCTAGGAGCTTGG	TGATCGCTGACAAGACTGTGG
Tfap2c	GGGCTTTTCTCTCTTGGCTGGT	TCCACACGTCACCCACACAA
Itgb1	ATGCCAAATCTTGCGGAGAAT	TTTGCTGCGATTGGTGACATT

Auxiliary Supplementary Material Legends

Movie S1, related to Figure 1B: XZ slices through an E6.5 Blimp1::mGFP embryo, moving from the distal to the proximal side of the egg cylinder, visualized using Imaris. Blimp1::mGFP (green), laminin (magenta), podocalyxin (gray) and DAPI (blue) are shown. Scale bar: 30 μm.
